# Synthesis of High Molecular Weight Stereo-Di-Block Copolymers Driven by a Co-Initiator Free Catalyst

**DOI:** 10.3390/polym14020232

**Published:** 2022-01-07

**Authors:** Carmen Moya-Lopez, Ivan Bravo, José A. Castro-Osma, David Chapron, Patrice Bourson, Christelle Vagner, Marianne Cochez, Nils Leoné, Agustín Lara-Sánchez, Carlos Alonso-Moreno, Daniel Hermida-Merino

**Affiliations:** 1 LMOPS, Centrale Supélec, Université de Lorraine, 57000 Metz, France; carmen.moya-lopez-pelaez@univ-lorraine.fr (C.M.-L.); david.chapron@univ-lorraine.fr (D.C.); patrice.bourson@univ-lorraine.fr (P.B.); christelle.vagner@univ-lorraine.fr (C.V.); marianne.cochez@univ-lorraine.fr (M.C.); 2 Centro Regional de Investigaciones Biomédicas, Unidad NanoCRIB, 02008 Albacete, Spain; Ivan.Bravo@uclm.es (I.B.); JoseAntonio.Castro@uclm.es (J.A.C.-O.); 3 Facultad de Farmacia de Albacete, Universidad de Castilla-La Mancha, 02008 Albacete, Spain; 4 Aachen-Maastricht Institute of BioBased Materials (AMIBM), Maastricht University, 6200MD Maastricht, The Netherlands; nils.leone@maastrichtuniversity.nl; 5 Departamento de Química Inorgánica, Orgánica y Bioquímica—Centro de Innovación en Química Avanzada (ORFEO-CINQA), Facultad de Ciencias y Tecnologías Químicas, Universidad de Castilla-La Mancha, 13071 Ciudad Real, Spain; Agustin.Lara@uclm.es; 6 Netherlands Organisation for Scientific Research (NWO), DUBBLE@ESRF BP CS40220, 38043 Grenoble, France

**Keywords:** stereo-diblock PLA copolymers, single site catalyst, ROP

## Abstract

Stereo-diblock copolymers of high molecular weight polylactide (PLA) were synthetized by the one pot-sequential addition method assisted by a heteroscorpionate catalyst without the need of a co-initiator. The alkyl zinc organometallic heteroscorpionate derivative (Zn(Et)(κ^3^-bpzteH)] (bpzteH = 2,2-bis(3,5-dimethylpyrazol-1-yl)-1-para-tolylethoxide) proved to assist in the mechanism of reaction following a coordination-insertion process. Kinetic studies along with the linear correlation between monomer and number average molecular weight (M_n_) conversion, and the narrow polydispersities supported the truly living polymerization character of the initiator, whereas matrix-assisted laser desorption/Ionization-time of flight (MALDI-TOF) studies showed a very low order of transesterification. The high stereo-control attained for the afforded high molecular weight derivatives was revealed by homonuclear decoupled ^1^H NMR spectra and polarimetry measurements. The nanostructure of the PLA derivatives was studied by both wide-angle X-ray scattering (WAXS) and differential scanning calorimetry (DSC) and the stereocomplex phase of the PLA stereo-diblock copolymers was successfully identified.

## 1. Introduction

Polyesters, and particularly polylactide (PLA), are largely investigated in the bioplastic field due to the low cost production, the remarkable physicochemical properties, and the biodegradability (under industrial compostable controlled conditions [1]). Furthermore, PLA is a semicrystalline polymer with bio-assimilate characteristics thanks to the hydrolysis of the polymer chain ester bond in physiological media, yielding lactic acid [2]. Hence, PLA is the bio-based polymer most widely employed in diverse industrial fields such as food handling, fibre manufacturing, textile industry or biomedical area [3]. However, the current PLA applicability is slightly inferior to conventional petroleum-based polymers due to their thermal resistance, mechanical properties and rate of crystallization [4]. Different strategies are being pursued to improve the physicochemical properties of PLA, such as the use of plasticisers or nucleating agents to enhance the detrimental slow crystallization rate [3,5,6]. Furthermore, the equimolar blend of poly-*L*-lactide (PLLA) and poly-*D*-lactide (PDLA) is an approach largely employed to generate stereocomplex (SC) crystallites as SC feature a melting point of 50 °C higher than its homocrystals (HC) counterparts due to the strong interactions between L- and D-lactyl unit sequences [5]. However, the SC crystallization of the blended enantiomers (PLLA and PDLA) diminishes for high molecular weight (HM_w_) PLA, and enantiomeric HC are obtained instead [6]. The synthesis of block copolymers formed by enantiomeric PLA strands is required to retain the SC crystallization in HM_w_ polymer derivatives. 

Previously, stereo-block-PLA derivatives have been achieved by the lengthening of building blocks on pre-polymers by a stepwise fashion [7], the extension of prepared PDLA and PLLA segments by reactive end-groups [8,9,10], or by solid-state polycondensation [11,12,13]. However, limitations on the preciseness and complexity of the desired polymer architecture are the main drawbacks of the prepolymer extension synthetic approaches (asymmetric blocks, high molecular weight distributions, transesterification reactions, etc.). Numerous efforts have been devoted over recent decades to the development of specific catalytic systems to promote the Ring-Opening Polymerization (ROP) of lactide (LA) attaining different tacticities under mild conditions to combine both the efficiency and polymerization control of ROP over polycondensation [2,14]. However, the advance of metalorganic catalysts for ROP that are able to retain the living character whilst assuring stereocontrol to avoid the common side reactions during the synthesis of HM_w_ polymers at mild experimental conditions has deterred the development of PLA derivatives with specific tacticities. Likewise, isotactic PLA synthesized from rac-LA by aluminium Schiff base catalytic systems was obtained with a high stereoselectivity [15]. Furthermore, heterotactic and syndiotactic PLA were also afforded from rac- and meso-LA, respectively, by catalysts featuring specific ligand characteristics, such as bulky substituents or chiral components [2,16,17,18]. In addition, the solvent interaction with the catalyst needs to be evaluated due to the strong influence on the final polymeric derivative tacticity as an enhancement of the steric hindrance around the metal centre might allow stereochemical control through a chain-end control mechanism [18]. Numerous efforts have also been devoted to synthesize PLA stereo block copolymers of HM_w_ following the one-pot sequential addition method. Particularly, the first PLA stereoblock of medium molecular weight was attained in the 90s; although, the increase in the reaction heterogeneity was found to decrease the living character of the catalyst [19]. In addition, detrimental side reactions were later ascertained to occur during polymerizations with homoleptic catalysts due to multiple nuclearities exhibited by the catalysts, which resulted in broad molecular weight distributions and lack of stereocontrol over the polymerization [15]. PLA stereoblock copolymers has also been achieved by the two-step polymerization of rac-LA combining stereo-selection and chiral ligand exchange [2], but requiring long polymerization times (up to 8 h). Recently, monomeric and highly active Zn and Mg complexes have been introduced to initiate the ROP of homo-chiral lactides to synthesize by a pot sequential addition HM_w_ stereo-n-block copolymers in the presence of alcohol as an activator. Stereo-di-block copolymers characterized with a high T_m_ and ΔH_m_ as well as very narrow polydispersities have been successfully synthesized by the one-pot sequential monomer addition method [20], suggesting the formation of desired SC crystals [21,22]. However, the development of novel robust catalysts able to attain HM_w_ stereo di-block-copolymers of PLA is still required to ascertain the mechanism of crystallization of the SC phase as well as to correlate the semicrystalline structure to its promising physicochemical properties. Although, slight structural imperfections and modest chain-length distribution are tolerated when designing block polymers in benefit of the most synthetically economical approach, such as the dispensability of the activator [23].

A potential efficient metalorganic catalytic system to attain HM_w_ stereo-block PLA by ROP should follow the coordination–insertion mechanism to avoid the termination step while maintaining a high activity for both L- and D-LA monomers during the synthesis as well as featuring a heteroleptic ligand to avoid transesterification reactions. In addition, a free-initiator catalyst will benefit the scalability of the synthesis to the industrial level since a more synthetically economical approach is achieved. Previously, a series of chiral scorpionate zinc alkyl complexes have been proved to catalyse as single-site living initiators through a truly living polymerization at mild conditions of both enantiopure L-LA and rac-LA monomers. Furthermore, the scorpionate zinc alkyl complexes featuring a myrtenal substituent on the alkoxide fragment produced enriched-heterotactic PLAs, due to the stereoselectivity exerted by the catalyst. The high conversions and stereocontrol achieved during the ROP of lactide monomers assisted by the chiral scorpionate zinc alkyl complexes have encouraged us to synthesise stereo-diblock-copolymers of PLA with HM_w_ due to their promising physicochemical properties. 

**Figure 1:**
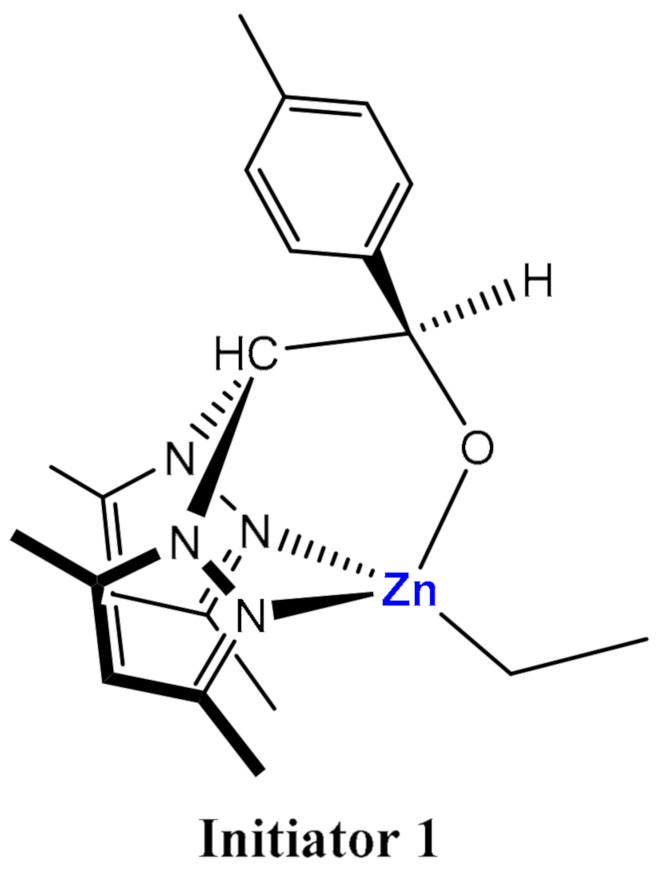
Chemical structure of Initiator **1**.

Herein, a series of PLA stereo-diblock-copolymers attaining different M_w_ were synthesized, through a Zn alkyl initiator with a p-tolyl substituent on the alkoxide fragment [Zn(Et)(κ^3^-bpzteH)] (bpzteH = 2,2-bis(3,5-dimethylpyrazol-1-yl)-1-para-tolylethoxide) (Initiator **1**, Figure 1) that acts as a single-site initiator at mild conditions and, notably, without the use of a co-initiator [24] for the first time to the best of our knowledge. The polymerization mechanism is discussed in detail by kinetic analysis as well as the characterization of the obtained polymer micro/nanostructures.

## 2. Materials and Methods

### 2.1. Materials

Solvents and reagents were commercially acquirable from Sigma-Aldrich (St. Louis, MO, USA) apart from L-lactide (L-LA) and D-lactide (D-LA) monomers that were purchased from Rex scientific. The lactide monomers were purified three times by sublimation and then stored in a glovebox at 4 °C. Toluene and tetrahydrofuran (THF) were pre-dried over sodium wire as well as distilled under nitrogen from sodium and subsequently stored over molecular sieves (3Å) in a glovebox. 

### 2.2. Synthesis

All the synthetic manipulations were performed under nitrogen, using standard Schlenk techniques. 

Synthesis of the initiator [Zn(Et)(κ^3^-bpzteH)] (bpzteH = 2,2-bis(3,5-dimethylpyrazol-1-yl)-1-para-tolylethoxide]. The synthesis of the Initiator 1 followed the previously published procedure [24]. Synthetic experimental details are disclosed in the Supplementary Information (SI) (Sections 1.1 and 1.2).

General procedure for solution polymerization of LA. Ring-opening polymerizations (ROP) were performed on a Schlenk line in a dried Schlenk flask equipped with a magnetic stirrer. The Schlenk tubes were loaded in the glovebox with the required amount of LA and Initiator **1**, separately, and dissolved in the appropriate amount of solvent (Table S1). Both LA and initiator Schlenk flasks were attached to the vacuum line and temperature equilibrium was ensured by stirring the solutions for 15 min in an oil bath. Monomer and catalyst solutions were poured together by a glass bent adaptor and polymerization onset times were measured from that point. Stereo-diblock copolymerizations were achieved by the addition of the second monomer upon the first monomer high conversion that was probed by monitoring reaction aliquots by ^1^H NMR. Methanol was used to terminate the reaction and precipitate out the polymer synthesized. The obtained polymer was collected by filtration, dried at room temperature, exposed to vacuum over 24 h in the Schlenk line, and stored upon characterization. The polymer derivatives experimental details and their respective characterization is detailed in the Supplementary Information (Figures S8–S26). 

Kinetic experiments. Reaction kinetic studies were conducted on the polymerizations of PLLA400 ([LA]_0_/[Zn]_0_ = 400; [LA]_0_ = 400 mM; [Zn]_0_ = 1 mM) at 90, 75 and 60 °C, PLLA and PDLA500 ([LA]_0_/[Zn]_0_ = 500; [LA]_0_ = 625 mM; [Zn]_0_ = 1.25 mM) at 90 °C, as well as L200:D200 ([LA]_0_/[Zn]_0_ = 400; [LA]_0_ = 400 mM; [Zn]_0_ = 1 mM) at 90 °C, to establish the reaction constant rate of each homopolymer enantiomer (Table S1, entries 1–6). The polymerization procedure was followed as previously described and aliquots were collected using a glass pipette at appropriate time intervals. All the volatiles in the aliquots were removed, and the residue was subjected to monomer conversion determination that was monitored by integration of methine resonances and its subsequent monomer against polymer ratio calculation in ^1^H NMR (CDCl_3_).

### 2.3. Instrumentation and Measurements

^1^H NMR, ^13^C NMR and homonuclear decoupled ^1^H NMR. Spectra were recorded at room temperature on a Varian Inova FT-400/500 spectrometer and referenced to the standard in the deuterated solvent with the relaxation time fixed to 4 s. The polymeric derivatives were dissolved in CDCl_3_ for the synthetic characterization as well as homonuclear decoupled ^1^H NMR and Toluene-d_8_ for variable temperature ^1^H NMR, respectively. Homonuclear decoupled ^1^H NMR spectroscopic analysis was conducted by applying the decoupling into the methyl region (δ = 1.5). The global spectrum deconvolution technique was performed when the multiplicity of the proton associated with the CH group of the polymer backbone corresponded to the tetrad signal (δ = 5.00–5.20), using both non-Bernouillan and Bernouillan statistics, further experimental calculations in Supplementary Information.

MALDI-TOF analysis. Matrix-assisted laser desorption/Ionization-time of flight (MALDI-TOF) spectra were acquired using a Bruker Autoflex II TOF/TOF spectrometer using dithranol (1,8,9-trihydroxyanthracene) and NaI as the matrix and the cationisation reagent, respectively. Samples co-crystallised with the matrix mixture in a ratio 100:1 on the probe were ionized in the positive reflector mode. External calibration was performed by using Peptide Calibration Standard II (covered mass range: 700–3200 Da) and Protein Calibration Standard I (covered mass range: 5000–17,500 Da).

GPC analysis. Molecular weight (M_n_, M_w_), as well as polydispersity index (PDI) of the afforded polymer derivatives, were determined from Gel permeation chromatography (GPC) using chloroform as eluent. The molecular weight characterization was performed with a Shimadzu Prominence-I LC-2030 equipped with a Shodex GPC KF-805L column (Shodex, Tokyo, Japan) and a Shimadzu RID-20A detector (Shimadzu, Kyoto, Japan). Analytical grade CHCl_3_ was used as the mobile phase at 40 °C, with a flow rate of 1 mL/min. GPC samples were prepared by dissolving ca. 5 mg of the corresponding polymer in 1.5 mL of solvent and left overnight under constant agitation. Thereafter, the samples were filtered over a 0.2 µm polytetrafluoroethylene (PTFE) syringe filter prior to its injection. Polystyrene with a molecular weight of 172 kDa was used as a reference to determine the overall molecular weight of the samples.

Optical rotation. The specific optical rotation (α) was measured in dichloromethane (DCM) at a concentration of 1mg/mL and 25 °C by a JASCO P-2000 WI using a beam wavelength of 589 nm.

DSC measurements. Differential scanning calorimetry (DSC) were performed using a TA Instruments Q2000 DSC (TA Instruments, New Castle, DE, USA) under nitrogen atmosphere. Temperature and heat flow were calibrated by using indium as a standard. DSC samples were prepared by loading around 3–5 mg of the polymer derivatives in Tzero Hermetic Aluminium pans (TA Instruments, New Castle, DE, USA). The thermal protocols consisted of a first heating ramp at 10 °C/min from 20 °C to 200 °C (homopolymers) or 230 °C (copolymers), respectively, and held for 2 min at the melt to erase thermal history. Samples were then cooled to 20 °C at a constant rate of 5 °C/min. The melting behaviour of all samples was observed by a new heating cycle to 200 or 230 °C, respectively, at a rate of 10 °C/min. The thermal transitions and their corresponding enthalpies such as the temperature and enthalpy of crystallization upon cooling (T_c_, ΔH_c_), temperature and enthalpy of cold crystallization upon heating (T_cc_, ΔH_cc_), as well as melting temperature and enthalpy of melting (T_m_, ΔH_m_) were calculated from the obtained thermograms. 

WAXS experiments. Wide-angle X-ray scattering (WAXS) experiments were performed at bl11 NCD-SWEET at ALBA, Cerdanyola del Vallès as well as DUBBLE (bm26) at ESRF in Grenoble. The wavelength of the X-ray beam was tuned to 12 Kev and the WAXS patterns were collected either with a Rayonix LX255-HS that features a pixel size of 40 µm^2^ × 40 µm^2^ and active area of 85 mm^2^ × 255 mm^2^ (h × v) or a Pilatus 300 kw featuring 1472 × 195 pixels with a pixel size of 172 µm^2^ × 172 µm^2^. The scattering angular range was calibrated using α-Al2O3 (alumina) as standard for the WAXS scattering angles. The WAXS patterns were reduced to 1D intensity profiles using bubble [21] as a function of the scattering vector (q = 4πsinθ/λ) and reported in arbitrary units WAXS profiles.

Computational Methodology. Computational Simulations were conducted by using Gaussian09 (Rev. C.01) software package (Gaussian 09). The theoretical calculations employed a hybrid density functional B3LYP method to minimise the geometries of all species involved. 6-31G (d) basic set was used to represent all atoms except Zinc (Zn). For Zn, LANL2DZ has successfully been applied to describe the geometry of complexes [22,25]. In addition, the solvent (toluene) was simulated using the Polarizable Continuum Model (PCM) methodology implemented in Gaussian09 package [26]. The initial estimations for the different intermediates were generated from the crystallographic structures of the dimer. Geometry at the selected level of theory (Table S2) was optimized for dimer structure, and then monomeric species were generated from them. Subsequently, an initial conformation analysis was carried out for all species to obtain the lowest energy conformation. The nature of the stationary points was assessed using the normal vibration frequencies calculated from the analytical second derivatives of the energy. The first-order saddle points—which are related to transition states—must show an imaginary value for the frequency associated with the eigenvector primarily describing the product formation step, whereas the real minima of the potential energy hypersurface—which are related to stable species—have to show positive real values for all the vibrational frequencies. Gibbs free energies used were obtained as the sum of electronic and thermal free energy at 298.15 K.

## 3. Results and Discussion

### 3.1. Characterization and Activity of the Initiator 1

The bioinspiring structure of the previously synthesized Zn complexes with multifunctional ligands reproducing the active centre of the metalloproteins together with their low cost confer them as great candidates to generate feasible biocompatible polymers. The acid character of the Zn complexes enhances the polymerization of cyclic esters as facilitates the coordination of the LA monomer to the metal. Likewise, the functionalization of the scorpionate ligand to tailor the electronic environment and the steric hindrance confers stability to the metallic complex as well as directs specific molecular conformation to coordinate with other ligands. Among the scorpionate derivative zinc alkyl complexes, the Initiator **1** (Figure 1), featuring an ethyl group as well as a scorpionate ligand with a tolyl (p-MeC_6_H_4_) substituent, was selected as a result of its potential higher activity aroused from the lower Zn-C bond, as well as the potential absence of hetero-enrichment due to the tolyl substituent [24]. The reactivity of the catalyst is crucial to one pot monomer sequential addition controlled by a living catalyst without the requirement of a co-initiator that potentially leads to detrimental chain transfer reactions [27]. Particular properties of metal complexes have been considered to design suitable catalysts for ROP of cyclic ester. Firstly, the metallic centre must present its highest stable oxidation state to avoid redox processes as well as hydrogen β-elimination reactions from the alkoxide of the growing polymer chain that potentially result in the loss of the catalytic activity. Moreover, the catalytic system fragment L_n_M should be inert with respect to the ligand exchange to prevent the formation of oligomeric alkoxides with the growing polymer chain. However, the activity of the Initiator 1 to ROP remained undescribed in the previous work [24] as well as its mechanism of reaction. Previously, an asymmetric dimeric conformation of the Initiator 1 featuring dinuclear complexes was found by X-ray diffraction analysis (XRD) in the solid state, whilst an equilibrium with the monomeric counterpart was not discarded in solution [24]. Furthermore, the existence of different enantiomers of Initiator 1 has been assessed by the addition of a chiral shift reagent (R)-(—)-(9-anthrul)-2,2,2-trifluoroethanol for ^1^H NMR solution experiment due to the existence of an asymmetric carbon in the ligand [24]. The presence in solution of only *meso*-enantiomer was confirmed by the appearance of only one signal for each proton. The crystalline structure manifests a tetrahedral arrangement for the metallic centre coordinated with a single pyrazol group and with a coordination κ-NN-μ-O to the ligand, whilst a distorted tetrahedral environment found for the Zn was found for a similar monomeric structure with a κ^3^-NNO coordination with the methyl group as alkyl substituent for the Zn complex [24]. The obtained ^1^H NMR chemical shifts along with the crystal structure suggested a tetrahedral environment for the zinc atom with the two pyrazole rings in *cis-* and *trans-* conformation in reference to the *para*-tolyl group for the monomeric monalkyl Zn complex [24]. However, the ^1^H NMR spectra is unable to ascertain the presence either the dimer or monomeric species in solution. 

**Figure 2:**
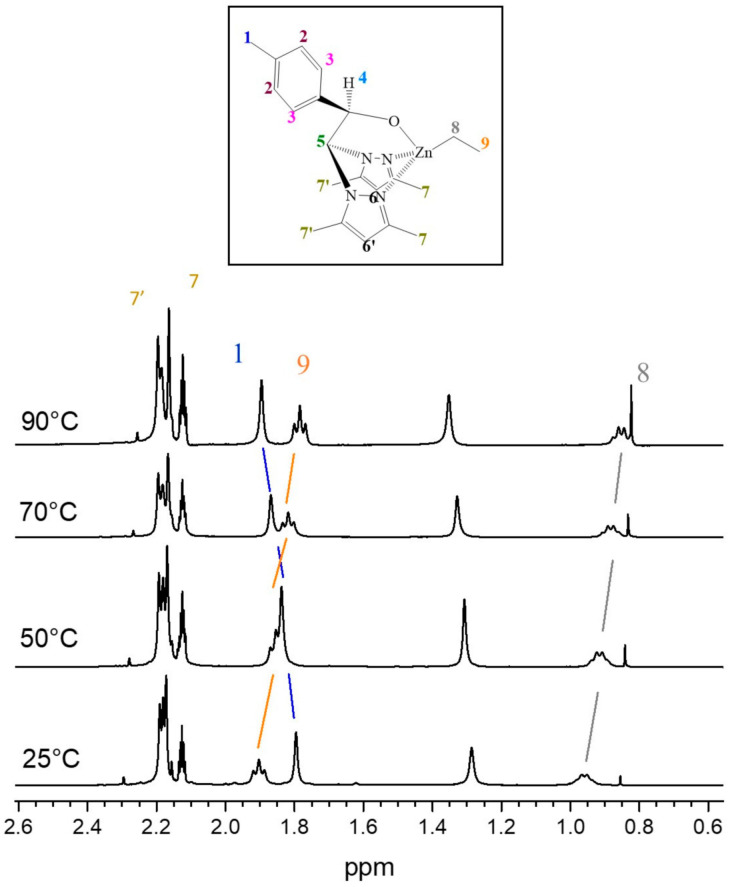
Variable temperature ^1^H NMR (500 MHz, Tol-d8, δ) spectra of Initiator **1**. ^1^H NMR (CDCl3, 297 K): 1.88-1.91 (t, 3H, H9); 1.79 (s, 3H, H1); 0.95-0.96 (q, 2H, H8).

Moreover, the truly active species of the metal complex during polymerization and its stability with increasing temperature was analysed by variable temperature ^1^H NMR (VT-^1^H NMR) of the catalyst in toluene (Figure 2) from 25 to 90 °C. Negligible multiplicity or width signal changes were observed upon heating, indicating the constant equivalence of all protons in solution. However, ethyl signals (δ 1.9 and 0.95) and p-tolyl methyl signal (δ 1.8) slightly shifted downfield and upfield (0.1 ppm) (Figure 2 and Figure S3), respectively, as the temperature increased, consistent with minor changes in the solvent–catalyst interactions or/and the molecular conformation of the ethyl substituent of the catalytic centre. The ^1^H NMR shifts at high temperature might suggest the monomeric species at high temperature. 

**Figure 3:**
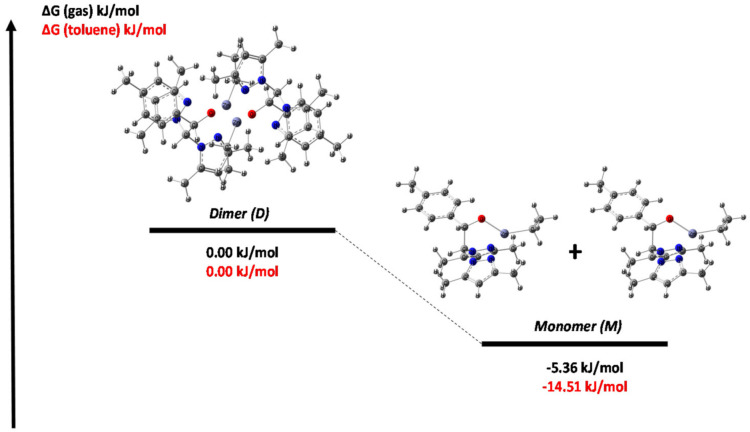
Energy profile for the dimeric and monomeric species. ΔG = 0 for the dimer in gas and toluene.

In addition, Densify Functional Theory (DFT) theoretical calculations were performed to provide an overall interpretation of the coexistence of monomeric and dimeric forms of the Initiator 1. Both the dimeric and monomeric conformations of the Initiator 1 were optimized and the energy profile of the most stable geometries in the process of rupture of the dimeric species was obtained (Figure 3). The yielded Gibbs free energy showed that the monomeric form was significantly more stable than the dimeric compound for both gas and solution (toluene) phases, affording ΔG of −5.36 and −14.51 kJ/mol, respectively, in agreement with VT-^1^H NMR analysis.

### 3.2. Polymerization Mechanism

Initiators following a coordination–insertion mechanism for the ROP of LA have attracted much attention for the generation of well-controlled HM_w_ PLA [27]. Most metallic initiators that follow a coordination–insertion mechanism require a co-initiator, such as alcohol, to favour the reaction equilibrium towards the truly active initiator [28]. However, the alcohol molecular structure (ex. Bifunctional, polyalcohols, etc.) leads to different chain architectures (linear or branched) that hamper the polymerization control and complicate the synthetic approach [27]. Moreover, the use of a co-initiator usually results in undesired transesterification reactions [27]. Consequently, the development of co-initiator free catalysts for ROP following a coordination–insertion mechanism is of considerable interest to control the molecular architecture. The Initiator 1 is included among the heteroscorpionate catalyst family that were previously proved to be effective initiators for ROP of rac-LA without a co-initiator requirement. 

**Figure 4:**
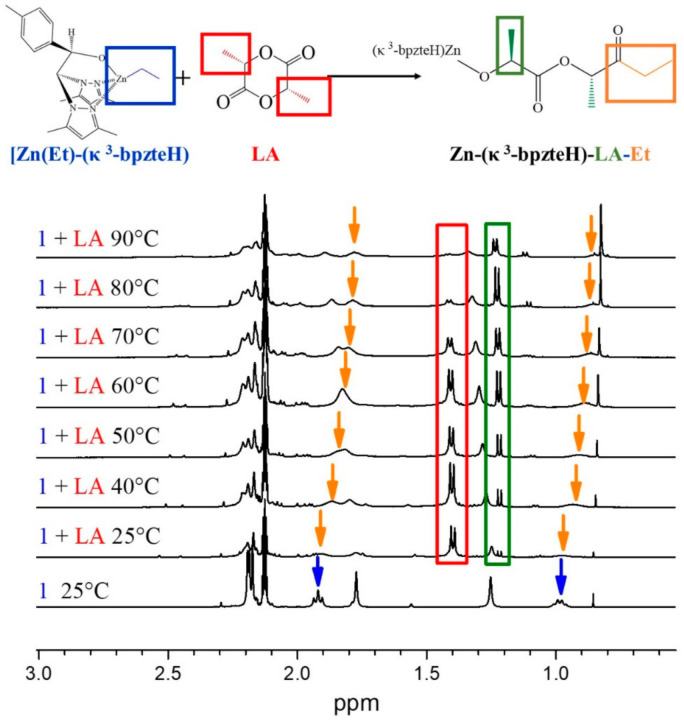
VT-^1^H NMR analysis of a [LA]/[Zn]:1 mixture.

The polymerization mechanism was assessed by following the ring-opening of the LA by the initiator 1 in a [LA]/[Zn] = 1 mixture, monitoring the key functional groups involved in the reaction by VT-^1^H NMR (Figure 4 and Figure S4). The coordination of the Zn atom to the carboxylic oxygen of LA and the subsequent insertion of the alkyl (Et) group formed an intermediate that was evidenced by the broadness of the ethyl signals (δ 1.9 and 0.95; blue and orange arrows) immediately after the addition of LA to the reaction mixture (δ 1.4 and 4.0–4; red box). Thereafter, the acyl–oxygen bond of LA is disrupted and the generated linear chain of LA turns into the alkoxide part of the catalyst (δ 1.2; green box), confirming the coordination–insertion mechanism.

**Figure 5:**
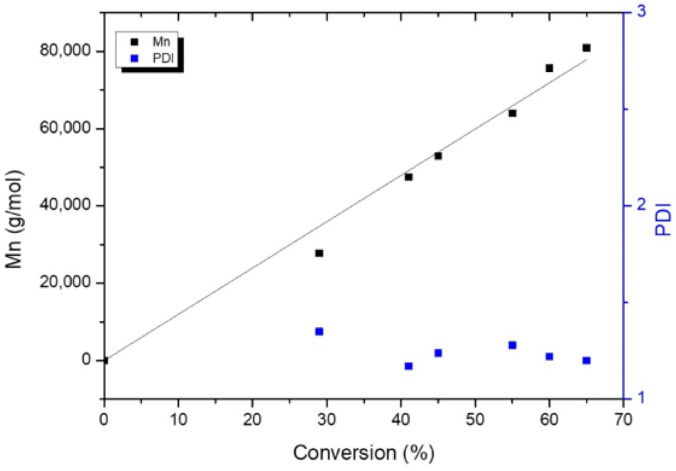
Plot of Mn and molecular weight distribution values (PDI) as a function of monomer conversion (%). [LA]_0_/[Zn]_0_ = 500, toluene, 90 °C.

The high level of chain control afforded by the Initiator 1 was further exemplified by the linear correlation between number average molecular weight (M_n_) and the percentage of conversion (R^2^ = 0.9958) that, along with the narrow PDI observed, evidenced the living character of the Initiator 1 (Figure 5). Moreover, the polymer chain architecture was evaluated by MALDI-TOF where a major population of linear polymers was found in a low molecular weight (M_w_) PLA derivative, with species separated by 144 Da and 72 Da (Figure S5). The formation of the shorter chain end (72Da) is generally ascribed to backbiting intramolecular transesterification reactions; although, the occurrence during the sample treatment cannot be ruled out. In addition, the M_w_ of the chemical fragments observed by MALDI-TOF (Figure S5) revealed the existence of a single family of polymer chains capped by –CH(CH_3_)OH and CH_3_(CH_2_)C(O)- termini, confirming that the ring-opening of LA occurs by the initial addition of the alkyl fragment to the LA monomer, following a nucleophilic route, with cleavage of the acyl–oxygen bond and subsequent monomer addition to the growing polymer chain. Likewise, the coordination–insertion mechanism proved by both VT-^1^H NMR and MALDI-TOF analysis allows the desired chain architecture control required to obtain SC PLA to be attained.

Furthermore, the stereocontrol exerted by Initiator **1** was ascertained for the ROP of a racemic LA mixture by assessing the presence of either the hetero enrichment or random insertion by homonuclear decoupling ^1^H NMR of poly(rac-LA) using both Bernouillan and non-Bernouillan statistics. The stereocontrol achieved by a particular initiator is expressed in terms of probability to generate certain stereosequences. P_m_ is known as the probability of isotactic enchainment while P_r_ is the probability of sindio/heterotactic enchainment [29]. Likewise, a random insertion is indicated when P_m_ = 0.5 whilst isotactic PLA is designated by P_m_ = 1, with the total probability expressed as P_m_ + P_r_ = 1 [30]. Stereocontrolled polymerization can be mediated by two distinct mechanisms, namely, chain-end control (CEC) and enantiomorphic site control (ESC). In the former case, the control of the chirality is known to be associated with the propagating chain end in which the transition state of the next monomer insertion defines the chirality of the next monomer unit to be incorporated into the growing chain. In contrast, for polymerizations mediated through an ESC mechanism, the chirality of the catalyst determines the chirality of the next monomer unit [30]. However, stereocontrol in the ROP of PLA is commonly achieved by CEC even when the initiator contains a chiral component. The deconvolution of the homodecoupled methine resonance of poly(rac-LA) derivative (Table S1, entry 15) revealed a five tetrad meso dyad in methane region (Figure S6), which was analysed by Bernouillan and non-Bernouillan statistics based on both CEC and ESC mechanism, respectively (Table S3). However, the probabilities calculated using non-Bernouillan statistics remained unresolved indicating that the stereocontrol exerted by Initiator **1** follows a CEC mechanism. Likewise, the P_m_ obtained by CEC for poly(rac-LA) was 0.52, in agreement with a completely atactic polymer. The homodecoupled methine resonance employing a CEC mechanism was applied to the afforded PLA derivatives (Section 2.1 in the SI).

### 3.3. Polymerization Kinetics

**Figure 6:**
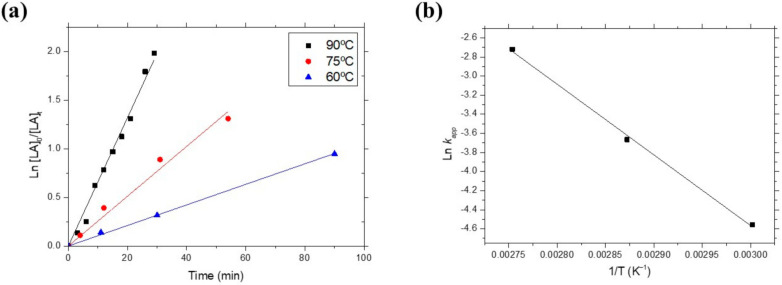
(**a**) First-order kinetic plots of L-LA polymerization in toluene at different temperatures. [LA]_0_/[Zn]_0_ = 400; [LA]_0_ = 400 mM; [Zn]_0_ = 1mM. (**b**) Plot of ln k_app_ versus T^−1^ for Initiator **1** in toluene with [LA]_0_/[Zn]_0_ = 400; [LA]_0_ = 400 mM; [Zn]_0_ = 1mM.

Polymerization rate constants were determined by solution kinetic studies at three different temperatures: 90, 75 and 60 °C. The order of reaction was calculated by plotting the ln([LA]_0_/[LA]_t_) versus reaction time where [LA]_0_ is the initial LA monomer concentration and [LA]_t_ is the LA concentration at a given time t (Figure 6a). The polymerization rate increased with increasing temperature, typical of Arrhenius systems (Table S4), with apparent rate propagation constants (k_app_) of the polymerization conducted in toluene one order higher than the k_app_ values found for polymerizations carried out in THF with the same family of initiators [24]. In addition, identical order of magnitude of k_app_ was found for polymerization previously performed in dichloromethane with other Zn-initiators [21]. The linearity of the plots corresponds to a first-order dependence with respect to the LA monomer, evidencing that termination reactions did not occur during polymerization. From the k_app_ values determined at different temperatures, the activation energy (E_a_) of the polymerization was deduced by fitting the Ln k_app_ versus T^−1^ according to the Arrhenius equation (Figure 6b). The E_a_ for the LA polymerization obtained, 61.57 kJ mol^−1^, was higher than the E_a_ data for organoaluminium initiators [31], which explains the slower polymerization kinetics observed.

In addition, the polymerization rate constant of both homopolymers, PLLA and PDLA, were compared to confirm the absence of hetero-enrichment yielded by the heteroscorpionate complexes counterparts previously described [24], which would be evidenced by a significant higher rate constant for one of the enantiomers. Generally, a catalyst featuring a chiral carbon will invariably result in stereocontrol of polymerization exerted by an ESC mechanism. However, the CEC mechanism becomes predominant in polymerizations using a chiral catalyst under harsh reaction conditions (i.e. high temperature and monomer concentration). In addition, a slight difference in the activation energy barrier between L- and D-LA monomers to form the coordinated complex catalyst–monomer might influence certain kinetic parameters when the polymerization approaches equilibrium, such as the monomer concentration at equilibrium that potentially would control the stereochemistry of the generated polymer. Likewise, L- and D-LA monomer conversions during two independent homopolymer reactions were monitored by ^1^H NMR, following the decrease in monomer concentration of both enantiomers during the PLA polymerizations. The modelled polymerization kinetics manifested a slightly higher rate for L-LA polymerization over the D-LA (entries 4 and 5 in Table S4) with a difference in monomer conversion of 10% after 45 min of reaction (85 and 95% for D- and L-LA, respectively). The similar polymerization rate constants for both D- and L-LA enantiomers obtained from kinetics experiments exhibited that the Initiator 1 is a potential catalyst for the synthesis of HM_w_ stereo-diblock copolymers.

### 3.4. Homochiral and Racemic Polymerization of Lactide

The ROP reaction conditions (time, temperature, and solvent) were optimized (Table 1 and entry 7–15; Table S1) from previously generated conditions to attain HM_w_ derivatives [24]. Mild reaction conditions at low [LA]/[cat] with 100 eq. of L-LA in THF at 50 °C prevented the onset of the polymerization even after 2 h of reaction (Entry 1, Table 1). The solvent exchange by a lower polarity solvent as toluene increased the conversion only to 5%. Only under more extreme conditions was the Initiator **1** able to promote the polymerization, converting 43 and 94% of monomer, in 2 h at 70 and 90 °C, respectively. HM_w_ polymers obtained with monomer conversions of 94% were obtained after reaction with 500 eq. of L-LA at 70 and 90 °C, in 120 and 45 min, respectively (Entry 6 and 7, Table 1). Moreover, monomer conversions higher than 85% were achieved for D- and rac-LA in 2 h (Entry 8 and 9, Table 1).

The obtained molecular weights were in good agreement with the calculated values, confirming the living character of the polymerization as well as the single-site catalyst mechanism, apart from polymerizations at ratio [LA]/[cat] = 100 (L1 and L2), which are likely related to a high k_prop_./k_inic_. ratio. Molecular weight distributions (PDI) were found to be narrow, although slightly increased for HM_w_ PLA derivatives due to both the steric hindrance and viscosity rise in the reaction mixture. Furthermore, polymerization of L-LA and D-LA yielded stereochemically pure PLA (Figures S8–S13), indicating that epimerization of either the monomer or the polymeric derivative was absent under the polymerization conditions. Likewise, the specific optical rotation (α) was evaluated from polarimetry measurements to corroborate the reaction stereocontrol (Table 1). The chiral purity may be estimated higher than 95% for all enantiopure derivatives (Table 1, entries 1–8) based on calculations from the reported value of (α) =173° at 598 nm for PLLA featuring perfect optical purity [32]. The specific optical rotation of poly(rac-LA) (Table 1, entry 9) was close to 0°, in agreement with the previous P_m_ analysis by deconvolution of the homonuclear decoupled ^1^H NMR spectrum.

### 3.5. Stereo-Block Copolymerization by Sequential Monomer Addition

Stereo-diblock co-polymers were obtained by the one-pot sequential addition method (Table 2, entries 16–19; Table S1). The ^1^H NMR monitored monomer consumption revealed conversions higher than 85% for both block sequences, which was in good agreement with the M_n_ determined by GPC. Likewise, the kinetic experiments of the addition of the second monomer yielded an equivalent k_app_ 6.2 ± 0.002 s^−1^ (Figure S7) with a first order of reaction, confirming the living character of polymerization upon the growth of the second block.

**Figure 7:**
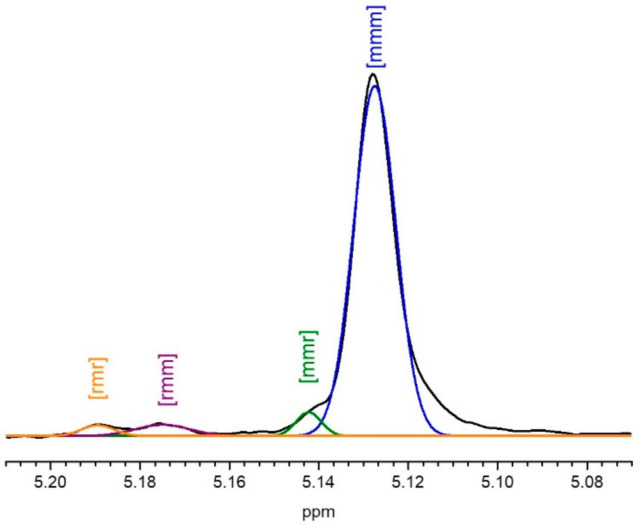
^1^H NMR spectra (500 MHz, 298 K, CDCl_3_) of the homodecoupled CH resonance of stereo-diblock copolymer 100L-b-100D (entry 2, Table 2).

Furthermore, the specific optical rotation (α) measured close to 0° for all samples, evidenced an L-LA/D-LA ratio of nearly one. The stereocontrol for the stereo-diblock co-polymer formation was assessed during the copolymerization by determining the tacticity of the copolymers obtained from deconvoluted homonuclear decoupling ^1^H NMR spectra using Bernouillan statistics. The deconvoluted methine spectral regions of the homonuclear decoupled ^1^H NMR spectrum of L100:D100 stereo-diblock co-polymer (Figure 7 as a representative example) were assigned to the appropriate tetrads following previous reports [33], and a Bernouillan statistical model was applied to the deconvoluted spectra. The obtained P_m_ values for the series of stereo-diblock copolymers (Table 2) confirmed the high stereocontrol achieved in the ROP for both blocks (>0.88), evidencing the almost full consumption of the two LA enantiomers as well as the isotactic stereo-diblock microstructure nature of the polymeric derivatives. 

**Figure 8:**
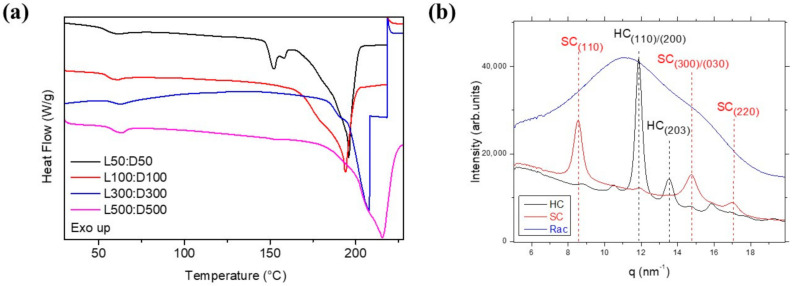
(**a**) DSC curves of stereo-diblock copolymers collected upon heating at 10 °C/min. (**b**) WAXS patterns acquired at room temperature. HC: homopolymer PLLA; SC: stereo-diblock copolymer; Rac: Poly(rac-lactide).

Additionally, DSC experiments were conducted on bulk polymers showing high melting temperatures for all generated stereo-diblock copolymers in the first DSC heating ramp (>193 °C), consistent with the SC crystalline phase [34,35] (Figure 8a), but slightly lower than T_m_ values previously reported [36,37]. Interestingly, the stereo-diblock copolymer attaining the lowest molecular weight (entry 1, Table 2) showed two melting transitions at 160 and 195 °C, likely related to homocrystal and stereocomplex phase respectively, as previously founded for blended SC PLA [5]. Furthermore, the stereocomplex crystalline phase was revealed by Wide-Angle X-Ray Scattering (WAXS) and compared to the corresponding enantiomeric and racemic counterparts (Figure 8b, 500L:500D, 1000 L, poly-rac 1000). Characteristic pattern of the SC crystalline phase [33,38,39] at (110), (300)/(030) and (220) (q (nm^−1^) = 8.5; 14.7 and 17, respectively) was observed for stereo-diblock copolymer, whilst the homopolymer counterpart (PLLA) showed the reflection of the HC phase at (110)/(200) and (203) (q (nm^−1^) = 11.9 and 13.6, respectively) (Figure 8b and Figure S27). Only amorphous halo was found for Poly(rac-LA) in agreement with the atactic microstructure obtained by P_m_ calculations (Figure 8b). The mechanism of crystallization of the novel stereo-diblock copolymers of PLA is under investigation and will be published in a future manuscript.

## 4. Conclusions

PLA stereocomplexation has typically been achieved by the blend of the PLA enantiomers with detrimental phase separation at a high molecular weight that diminishes the promising physicochemical properties featured by the SC phase. Particularly, the enhancement of the mechanical properties, as well as the thermal and hydrolysis degradation resistance of PLA derivatives, is still required, which could potentially be attained by the generation of stereo-diblock PLA copolymers. One of the strategies relies on the one-pot sequential addition of LA monomers to a living initiator to yield tailor-made copolymers. However, the one-pot sequential approach is very limited due to the unavailability of initiators exhibiting: (i) a living character, to obtain HM_w_ PLA derivatives as the monomer/initiator ratio increases; (ii) reactivity to polymerize both L- and D-LA monomers; (iii) similar rate constant for both L- and D-LA polymerizations, to achieve stereo-block copolymers in a relatively short time; (iv) negligible transesterifications reactions to control the final PLA-derivative architecture [15]; and (v) a simple synthetic approach to potentially scale the process to the industrial level [23].

Herein, we report the first organometallic initiator able to generate high molecular weight stereo-diblock copolymers of PLA following a coordination–insertion mechanism without the use of a co-initiator. The reaction conditions were optimized to attain SC-PLA exhibiting high molecular weight and relatively narrow PDI. Kinetic experiments confirmed the first-order reaction and similar rate constant of the polymerization for each block, and the homonuclear decoupled ^1^H NMR spectra and specific optical rotation that the fine stereocontrol was achieved under likely chain-end control and minimal transesterification reactions. The SC phase formation of the stereo-diblock copolymers was confirmed by DSC and WAXS analysis as well as by a high degree of tacticity consistent with conversion data acquired by ^1^H NMR.

The low toxicity and price offered by Zn-based catalysts offer the possibility to tailor the properties of novel biobased polymeric materials particularly crucial for biomaterials with nanomedicine applications, such as drug delivery systems or 3D scaffolds for tissue engineering. An in-depth study of the physical properties of the stereo-diblock copolymers generated is currently in progress to understand the structure–property relationship to tailor the final material applications.

## Figures and Tables

**Table 1:** Polymerizations of L-, D-, and rac-LA.

Entry	Sample	[LA]/[cat]	Temperature (°C)	Time (min)	Conv. (%)	M_n (theor_._)_ (Da) ^a^	M_n (exp_._)_ (Da) ^b^	PDI	(α) ^c^
1	PLLA100	100	50	120	0	0	-	-	
2	PLLA100	100	50	120	5	720	-	-	
3	PLLA100 (L1)	100	70	60	43	6192	12,824	1.36	−159.4
4	PLLA100 (L2)	100	90	60	94	13,536	30,055	1.62	−171.4
5	PLLA500 (L3)	500	90	45	94	67,680	62,564	1.84	−172.2
6	PLLA500 (L4)	500	70	90	76	54,720	42,880	1.7	−162.7
7	PLLA500 (L5)	500	70	120	94	67,680	48,253	2.19	−168.4
8	PDLA500 (D1)	500	70	90	85	61,200	41,292	1.78	154
9	rac-PLA500 (Rac1)	500	70	120	90	64,800	14,212	1.66	−1.1

**Table 2:** Stereo-block copolymerizations of L-LA and D-LA.

Entry	Sample	Time (min) ^a^	Conv. (%)	M_n (theor.)_ (Da) ^b^	M_n (exp.)_ (Da) ^c^	T_m_ (°C) ^d^	ΔH_m_ (J/g) ^e^	PDI	P_m_ ^f^	(α) ^g^
1	(L50:D50)	50	95	13,680	17,007	160–195.85	4.66–52.7	1.93	0.88	2.47
2	(L100:D100)	60	95	27,360	35,253	193.93	44.91	2.25	0.90	1.07
3	(L300:D300)	100	97	83,808	63,853	213.34	58.65	1.69	0.95	0.69
4	(L500:D500)	120	85	122,400	64,976	215.33	57.03	2.31	0.92	1.65

Polymerization conditions: 25 µmol of Initiator **1**, toluene as a solvent at 90 °C. ^a^ 30–60 min was maintained between each monomer addition, depending on the monomer loading. ^b^ Theoretical Mn = (monomer/Initiator 1) × (% conversion) × (Mw of LA). ^c^ Determined by GPC relative to polystyrene standards in chloroform. ^d,e^ The Tm and ΔHm was the melting temperature and enthalpy during the first heating. ^f^ Probability of finding meso dyads calculated from homonuclear decoupling ^1^H NMR spectra after deconvolution; calculations are based on CEC statistics. ^g^ Specific optical rotation ((α)PLLA = −173°) [32].

## Data Availability

No applicable.

## Supplementary Materials

The following are available online at https://www.mdpi.com/article/10.3390/polym14020232/s1, Figure S1: 1H NMR spectrum of bpm; Figure S2: 1H NMR spectrum of (Zn(Et)(κ3-bpzteH); Figure S3: VT- 1H NMR spectrum of (Zn(Et)(κ3-bpzteH); Figure S4: VT- 1H NMR spectrum of [LA]/[cat]=1 in the CH region; Figure S5: Maldi-Tof spectrum of PLLA20; Figure S6. 1H NMR spectra (500 MHz, 298 K, CDCl3,) of the homodecoupled CH resonance of poly(rac-36 lactide); Figure S7: First-order kinetic plot of the second block polymerization reaction of L200:D200 at 90 °C; Figure S8: 1H NMR spectrum of L1; Figure S9: 1H NMR spectrum of L2; Figure S10: 1H NMR spectrum of L3; Figure S11: 1H NMR spectrum of L4; Figure S12: 1H NMR spectrum of L5; Figure S13: 1H NMR spectrum of D1; Figure S14: 1H NMR spectrum of Rac1; Figure S15: 1H NMR spectrum of L50:D50; Figure S16: 1H NMR spectrum of the homodecoupled CH resonance of L50:D50; Figure S17: 13C NMR spectrum (500 MHz, 298 K, CDCl3) of L50:D50; Figure S18: 1H NMR spectrum of L100:D100; Figure S19: 1H NMR spectrum of the homodecoupled CH resonance of L100:D100; Figure S20: 13C NMR spectrum (500 MHz, 298 K, CDCl3) of L100:D100; Figure S21: 1H NMR spectrum of L300:D300; Figure S22: 1H NMR spectrum of the homodecoupled CH resonance of L300:D300; Figure S23: 13C NMR spectrum (500 MHz, 298 K, CDCl3) of L300:D300; Figure S24: 1H NMR spectrum of L500:D500; Figure S25: 1H NMR spectrum of the homodecoupled CH resonance of L500:D500; Figure S26: 13C NMR spectrum (500 MHz, 298 K, CDCl3) of L500:D500; Figure S27: WAXS patterns acquired at room temperature; Table S1: Polymerizations catalysed by Initiator 1; Table S2: Z-matrices for DFT calculation; Table S3. Tetrad probabilities of ESC and CEC mechanisms based on non-Bernouillan and Bernouillan 38 statistics; Table S4. Rate constants.
