# Supplementary material for: Synthesis of High Molecular Weight Stereo-Di-Block Copolymers Driven by a Co-Initiator Free Catalyst
Source: Polymers (Basel). 2022 Jan 7;14(2):232. doi: 10.3390/polym14020232 (PMC8779727; doi:10.3390/polym14020232)
Supplement: Supplementary file 1 [file polymers-14-00232-s001.zip › SI_Polylactide_stereoblock_Polymers_Final_version.pdf]

polymers

MDPI

Article

Synthesis of high molecular weight stereo-di-block copolymers driven by a co-initiator free catalyst

Carmen Moya-Lopez[1], Ivan Bravo[2,3], J.A. Castro-Osma[2,3], David Chapron[1], Patrice Bourson[1], Christelle Vagner[1], Marianne Cochez[1], Nils Leoné[4], Agustín Lara-Sánchez[5], Carlos Alonso-Moreno[2,3]*and Daniel Hermida-Merino[6]*

[1] LMOPS, CentraleSupelec, Université de Lorraine, 57000 Metz, France.

[2] Centro Regional de Investigaciones Biomédicas, Unidad NanoCRIB, Albacete-02008, Spain.

[3] Universidad de Castilla-La Mancha. Facultad de Farmacia de Albacete, Albacete-02008, Spain.

[4] Aachen-Maastricht Institute of BioBased Materials (AMIBM), Maastricht University, 6200MD Maastricht, The Netherland.

[5] Departamento de Química Inorgánica, Orgánica y Bioquímica-Centro de Innovación en Química Avanzada (ORFEO-CINQA), Facultad de Ciencias y Tecnologías Químicas, Universidad de Castilla-La Mancha,13071 Ciudad Real, Spain.

[6] Netherlands Organisation for Scientific Research (NWO), DUBBLE@ESRF BP CS40220, 38043 Grenoble, France.

*Correspondence: hermidam@esrf.fr; Tel.: +33(0)476882375; carlos.amoreno@uclm.es, Tel.: +34926295300, Ext. 3499

Citation: Lastname, F.; Lastname, F.; Lastname, F. Title. *Polymers* **2022**, *13*, x. https://doi.org/10.3390/xxxxx

Academic Editor: Firstname Lastname
Received: date
Accepted: date
Published: date

Publisher's Note: MDPI stays neutral with regard to jurisdictional claims in published maps and institutional affiliations.

Copyright: © 2022 by the authors. Submitted for possible open access publication under the terms and conditions of the Creative Commons Attribution (CC BY) license (http://creativecommons.org/licenses/by/4.0/).

Supplementary Information

Table of Contents

Table S1: Polymerizations catalysed by Initiator 1 4

1. Characterization and activity of the catalyst **4**

1.1 Synthesis of the 2,2-bis((3,5-dimethyl-pirazol-yl)-1-para-tolylethoxide) (bpzteH) 4

Figure S1: ^{1}H NMR spectrum of bpm 5

1.2 Synthesis of [Zn(Et)]-[2,2-bis((3,5-dimethyl-pirazol-yl)-1-para-tolylethoxide)] (Zn(Et)(κ³-bpzteH) 5

Figure S2: ^{1}H NMR spectrum of (Zn(Et)(κ³-bpzteH) 6

Figure S3: VT- ^{1}H NMR spectrum of (Zn(Et)(κ³-bpzteH) 7

Table S2: Z-matrices for DFT calculation 7

2. Polymerization mechanism **13**

Figure S4: VT- ^{1}H NMR spectrum of [LA]/[cat]=1 in the CH region 13

Figure S5: Maldi-Tof spectrum of PLLA20 14

2.1 Calculation of Pm values of Racemic and stereo-diblock copolymers of PLA 14

Figure S6. ^{1}H NMR spectra (500 MHz, 298 K, CDCl3,) of the homodecoupled CH resonance of poly(rac-lactide) 15

Table S3. Tetrad probabilities of ESC and CEC mechanisms based on non-Bernouillan and Bernouillan statistics 15

3. Polymerization kinetics **15**

Table S4. Rate constants 15

Figure S7: First-order kinetic plot of the second block polymerization reaction of L200:D200 at 90°C 16

4. Homochiral and racemic polymerization of lactide **16**

4.1 Synthesis of L1: Homopolymer synthesis 16

Figure S8: ^{1}H NMR spectrum of L1 17

4.2 Synthesis of L2 17

Figure S9: ^{1}H NMR spectrum of L2 18

4.3 Synthesis of L3 18

Figure S10: ^{1}H NMR spectrum of L3 19

4.4 Synthesis of L4 19

Figure S11: ^{1}H NMR spectrum of L4 20

4.5 Synthesis of L5 20

Figure S12: ^{1}H NMR spectrum of L5 21

4.6 Synthesis of D1 21

Figure S13: ^{1}H NMR spectrum of D1 22

4.7 Synthesis of Rac1 22

Figure S14: ^{1}H NMR spectrum of Rac1 23

5. Stereo-block copolymerization by sequential monomer addition **22**

5.1 Synthesis of L50:D50 23

Figure S15: ^{1}H NMR spectrum of L50:D50 24

Figure S16: ^{1}H NMR spectrum of the homodecoupled *CH* resonance of L50:D50 24

Figure S17: ^{13}C NMR spectrum (500 MHz, 298 K, $CDCl_3$) of L50:D50 25

5.2 Synthesis of L100:D100 25
Figure S18: ^{1}H NMR spectrum of L100:D100 26
Figure S19: ^{1}H NMR spectrum of the homodecoupled *CH* resonance of L100:D100 26
Figure S20: 13C NMR spectrum (500 MHz, 298 K, CDCl3) of L100:D100 27

5.3 Synthesis of L300:D300 27
Figure S21: ^{1}H NMR spectrum of L300:D300 28
Figure S22: ^{1}H NMR spectrum of the homodecoupled *CH* resonance of L300:D300 28
Figure S23: ^{13}C NMR spectrum (500 MHz, 298 K, $CDCl_3$) of L300:D300 29

5.4 Synthesis of L500:D500 29
Figure S24: ^{1}H NMR spectrum of L500:D500 30
Figure S25: ^{1}H NMR spectrum of the homodecoupled *CH* resonance of L500:D500 30
Figure S26: ^{13}C NMR spectrum (500 MHz, 298 K, $CDCl_3$) of L500:D500 31
Figure S27: WAXS patterns acquired at room temperature 31

Entry	Sample	[LA]/[cat]	Solvent	Temperature (°C)	Time (min)	Conversion (%)	$M_{n\ (theor.)}$ (Da)[a]	$M_{n\ (exp.)}$ (Da)[b]	PDI	[α][c]
1	PLLA400	400	Toluene	60	90	90	51840	47539	1,97	-
2	PLLA400	400	Toluene	75	50	89	51264	33861	1,78	-
3	PLLA400	400	Toluene	90	30	90	51840	43348	1,85	-
4	PLLA500	500	Toluene	90	45	68	48960	62564	1,84	-
5	PDLA500	500	Toluene	90	45	61	43920	33442	1,96	-
6	L200:D200	400	Toluene	90	60	88	50688	32958	2,2	-
7	PLLA100	100	THF	50	120	0	0	-	-	-
8	PLLA100	100	Toluene	50	120	5	720	-	-	-
9	PLLA100 (L1)	100	Toluene	70	60	43	6192	12824	1,36	-159,4
10	PLLA100 (L2)	100	Toluene	90	60	94	13536	30055	1,62	-171,4
11	PLLA500 (L3)	500	Toluene	90	45	94	67680	62564	1,84	-172,2
12	PLLA500 (L4)	500	Toluene	70	90	76	54720	42880	1,77	-162,7
13	PLLA500 (L5)	500	Toluene	70	120	94	67680	48253	2,19	-168,4
14	PDLA500 (D1)	500	Toluene	70	90	85	61200	41292	1,78	154
15	rac-PLA500 (Rac1)	500	Toluene	70	120	90	64800	14212	1,66	-1,1
16	(L50:D50)	100	Toluene	90	50	95	13680	17007	1,93	-2.47
17	(L100:D100)	200	Toluene	90	60	95	27360	35253	2,25	-1.07
18	(L300:D300)	600	Toluene	90	100	97	83808	63853	1,69	-0.69
19	(L500:D500)	1000	Toluene	90	120	85	122400	64976	2,31	-1.65

Table S1. Polymerizations catalysed by Initiator **1**. Polymerization conditions: 25μmol of initiator. [a]Theoretical M_n = (monomer/initiator) x (% conversion) x (M_w of LA). [b]Determined by GPC relative to polystyrene standards in chloroform. [c]Specific optical rotation ($[\alpha]_{PLLA}$=-173°) [30].

1. Characterization and activity of the catalyst

1.1 Synthesis of the 2,2-bis((3,5-dimethyl-pirazol-yl)-1-para-tolylethoxide) (bpzteH)

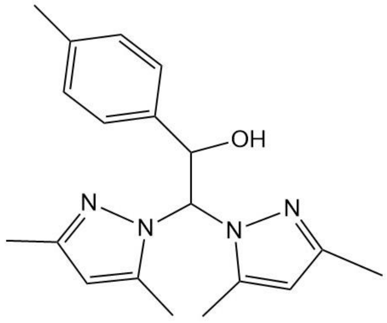

The ligand (bpzteH) was synthesized via the following procedure: In a 250 mL Schlenk tube, Bis 3,5-(dimethyl-pirazol-yl)-methylene (bpm) (3 g, 14.7 mmol) was dissolved in dry THF (70 mL) and then cooled to -78°C. A 2.5 M solution of Bu_nLi (6.47 mL, 16.17 mmol) in hexane was added to the bpm solution and stirred for 1h. The resulting mixture was added dropwise to a cooled (-10°C) solution of p-tolyl (1.86 g, 15.43 mmol). The reaction mixture was allowed to warm up to ambient temperature and was stirred for 1h. The obtained product was hydrolyzed with saturated aqueous NH_4Cl

(15 mL). The organic layer was extracted, dried over $MgSO_4$, filtered, and the solvent was removed in vacuum to yield the product as a yellow oil, which was titrated with hexane to afford the pure product as a yellow solid (1.54 g, 75%)). ^{1}H NMR (500 MHz, CDCl3, 297 K): δ 7.25-7.23 (d, 2H, H3), 6.91-6.88 (d, 2H, H2), 6.28-6.26 (d, 1H, H6), 5.90-5.89 (d, 1H, H6), 5.59 (s, 1H, H4), 5.44 (d, 1H, H5), 2.14-1.55 (m, 12H, H7), 1.44 (s, 3H, H1).

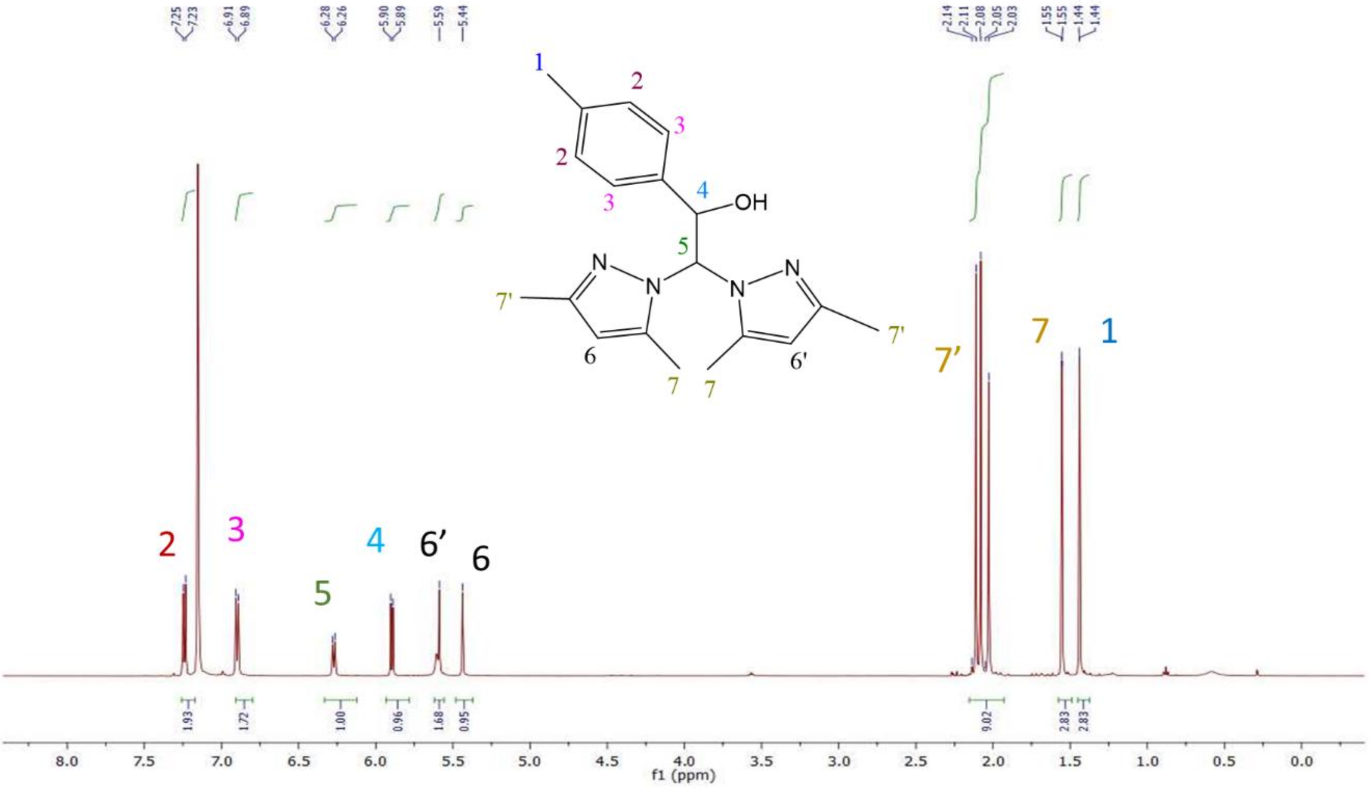

Figure S1: ^{1}H NMR spectrum (500 MHz, 298 K, $CDCl_3$) of the ligand 2,2-bis((3,5-dimethyl-pirazol-yl)-1-para-tolylethoxide) (bpzteH).

1.2 Synthesis of [Zn(Et)]-[2,2-bis((3,5-dimethyl-pirazol-yl)-1-para-tolylethoxide)] (Zn(Et)(κ3-bpzteH)

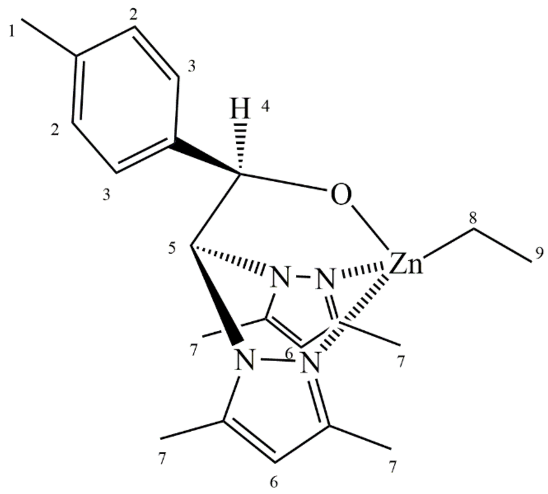

The catalyst (Zn(Et)(κ3-bpzteH) was synthesized by the following procedure: The ligand (bpzteH) was dissolved in dry toluene and $Zn(Et)_2$ was added through a cannula to the solution placed on ice-bath (0°C). The resulted solution was stirred for 2h at room temperature and the solvent was removed in vaccum. Yield : 1,54g, 75%. ^1H NMR (500 MHz, CDCl3, 297 K): δ 6.97-6.95 (d, 2H, H3), 6.89-6.87 (d, 2H, H2), 5.90-5.88 (d, 2H, H6), 5.68 (d, H, H4), 5.41 (d, 1H, H5), 2.34-2.24 (s, 12H, H7), 1.52 (s, 3H, H1), 1.40-1.36 (t, 3H, H9), 0.45-0.39 (q, 2H, H8).

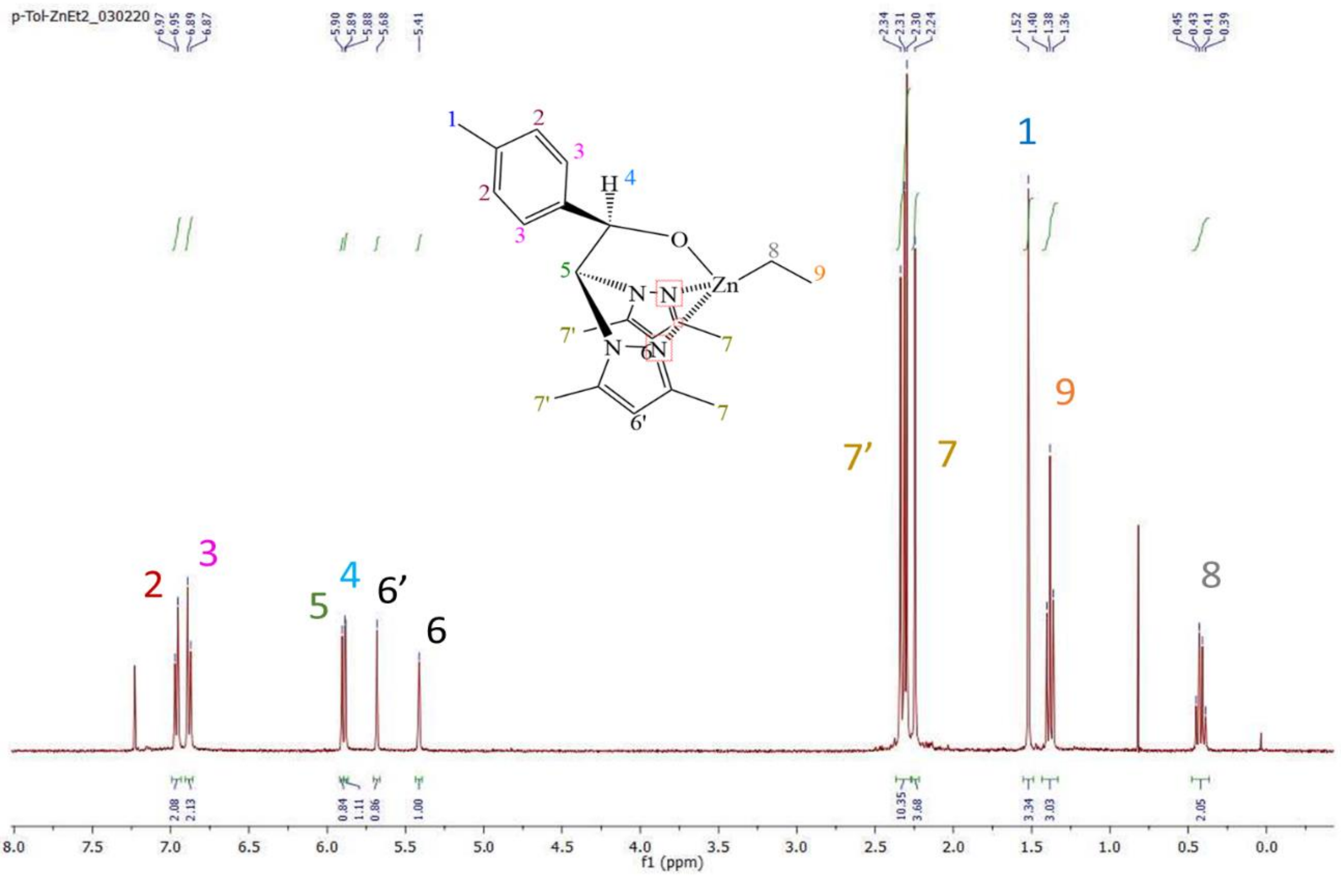

Figure S2: ^1H NMR spectrum (500 MHz, 298 K, $CDCl_3$) of (Zn(Et)(κ3-bpzteH).

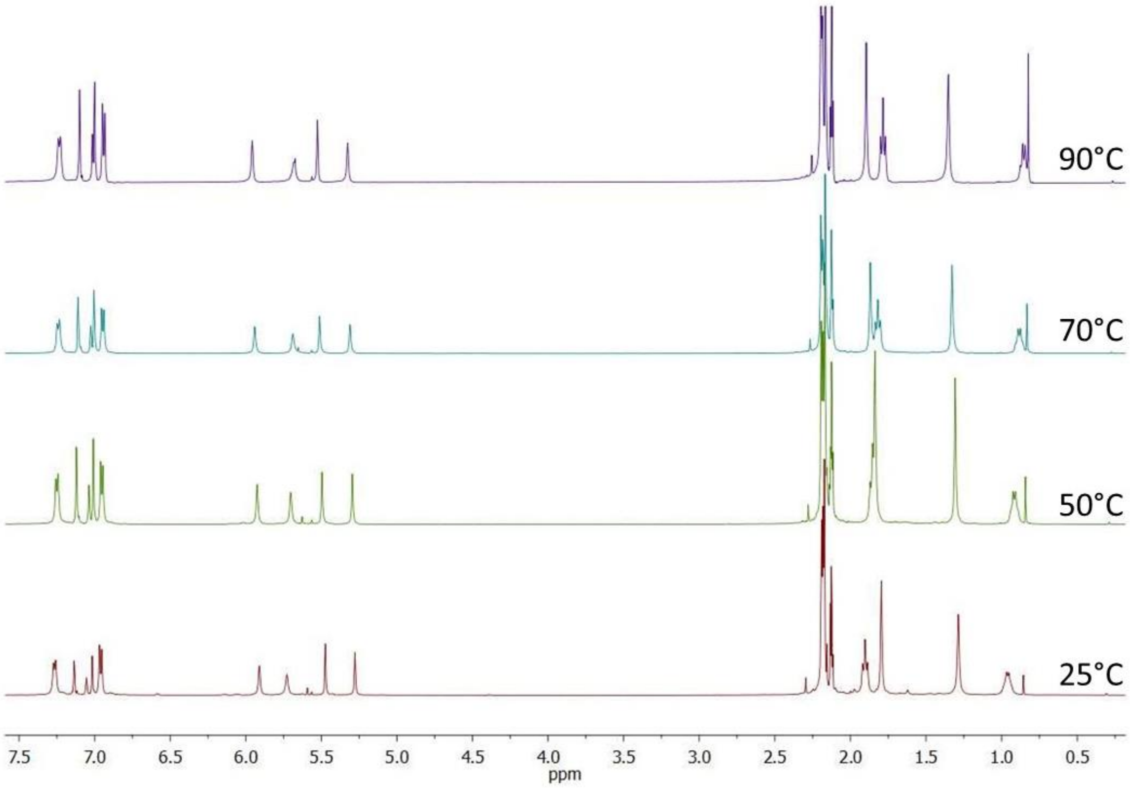

Figure S3: VT- ^{1}H NMR spectrum (500 MHz, 298 K, $CDCl_3$) of (Zn(Et)(κ3-bpzteH). ^{1}H NMR (Told8, 298 K): δ 7.25-7.27 (d, 2H, H3), 6.95-6.96 (d, 2H, H2), 5.910 (s,1H, H6′), 5.728 (s,1H, H6), 5.473 (d, H, H4), 5.27 (d, 1H, H5), 2.11-2.19 (s, 12H, H7), 1.79 (s, 3H, H1), 1.88-1.91 (t, 3H, H9), 0.95-0.96 (q, 2H, H8). ^{1}H NMR (Told8, 348 K): δ 7.24-7.25 (d, 2H, H3), 6.94-6.96 (d, 2H, H2), 5.92 (s,1H, H6′), 5.70 (s,1H, H6), 5.49 (d, H, H4), 5.29 (d, 1H, H5), 2.05-2.25 (s, 12H, H7), 1.83 (s, 3H, H1), 1.85-1.87 (t, 3H, H9), 0.90-0.92 (q, 2H, H8). ^{1}H NMR (Told8, 368 K): δ 7.23-7.24 (d, 2H, H3), 6.94-6.95 (d, 2H, H2), 5.94 (s,1H, H6′), 5.68 (s,1H, H6), 5.51 (d, H, H4), 5.31 (d, 1H, H5), 2.11-2.19 (s, 12H, H7), 1.86 (s, 3H, H1), 1.80-1.81 (t, 3H, H9), 0.87-0.89 (q, 2H, H8). ^{1}H NMR (Told8, 388 K): δ 7.22-7.24 (d, 2H, H3), 6.93-6.94 (d, 2H, H2), 5.95 (s,1H, H6′), 5.68 (s,1H, H6), 5.52 (d, H, H4), 5.32 (d, 1H, H5), 2.11-2.25 (s, 12H, H7), 1.89 (s, 3H, H1), 1.76-1.80 (t, 3H, H9), 0.84-0.87 (q, 2H, H8).

Table S2. Z-matrices for DFT calculations.

**Monomer (Toluene)**	**Dimer (Toluene)**	**Monomer (gas)**	**Dimer (gas)**
Zn 1.70263700 -	0 1	Zn -2.57890000	Zn 11.44420000
1.23998700 -0.49703200	Zn 0.48583200	1.96020000 -1.20200000	6.00550000 5.15830000
N 2.18148500	0.96921000 1.20056800	N -3.05530000 -	N 12.84750000
0.96859500 -0.31739100	N 1.04675900	0.23030000 -0.92490000	4.75770000 6.03750000
N 1.08538800	2.63034200 -0.19517100	N -1.96970000 -	N 14.13040000
1.74432700 -0.08444600	N 2.07671000	0.98470000 -0.59890000	4.68580000 5.57390000
N 0.51639300 -	2.42517700 -1.06250600	N -1.47500000	N 12.62300000
0.77974100 1.37114300	N 3.13238200	1.60580000 0.74630000	4.36690000 3.13160000
N -0.30224500	1.43255500 1.60874700	N -0.63890000	N 13.95810000
0.30297900 1.24655600	N 3.77367500	0.53080000 0.70530000	4.62570000 3.18360000
O 0.10016400 -	1.57319900 0.41695000	O -0.92630000	O 12.94530000
0.75258700 -1.53415800		1.45260000 -2.11980000	7.23410000 4.66720000

C 1.41211500<br>3.07151700 -0.05922600	O 1.32044600 -<br>0.29786300 -0.18096200	C -2.29560000 -<br>2.30880000 -0.50780000	C 14.91280000<br>3.96140000 6.42470000
C 2.77638800<br>3.13788400 -0.28292100	C 2.08690900<br>3.36953600 -2.05111700	C -3.64840000 -<br>2.39490000 -0.78690000	C 14.09780000<br>3.57220000 7.45340000
H 3.37539600<br>4.03646600 -0.32743100	C 1.01411500<br>4.20603800 -1.79952200	H -4.24380000 -<br>3.29690000 -0.80330000	H 14.34470000<br>3.06680000 8.19390000
C 3.21534600<br>1.80438000 -0.43572400	H 0.71647900<br>5.06349000 -2.38635800	C -4.08190000 -<br>1.07460000 -1.03890000	C 12.83110000<br>4.07570000 7.18000000
C 0.42130000<br>4.17019500 0.16724900	C 0.39510000<br>3.71003600 -0.63220200	C -1.31380000 -<br>3.38710000 -0.17070000	C 16.35030000<br>3.66530000 6.17190000
H -0.07670100<br>4.08288600 1.14048900	C 3.09465700<br>3.41108400 -3.15755500	H -0.84450000 -<br>3.22810000 0.80810000	H 16.45160000<br>3.30030000 5.29020000
H 0.93773100<br>5.13296000 0.14518000	H 4.11646200<br>3.56098100 -2.78717500	H -1.83010000 -<br>4.34970000 -0.13660000	H 16.66490000<br>3.03030000 6.81980000
H -0.35547500<br>4.19225700 -0.60660800	H 2.86154400<br>4.24409800 -3.82562600	H -0.51310000 -<br>3.46720000 -0.91630000	H 16.86100000<br>4.47480000 6.24390000
C 4.59937100<br>1.29073300 -0.69125400	H 3.08559000<br>2.49315100 -3.75754400	C -5.45290000 -<br>0.57940000 -1.38530000	C 11.54500000<br>3.91570000 7.92210000
H 4.99815700<br>1.68396600 -1.63356900	C -0.81785600<br>4.22596400 0.07969800	H -5.81420000 -<br>1.03650000 -2.31390000	H 11.24500000<br>4.77370000 8.23070000
H 5.28661100<br>1.59386900 0.10710100	H -1.73360300<br>3.76028600 -0.30162600	H -6.17320000 -<br>0.82570000 -0.59630000	H 11.67910000<br>3.33420000 8.67420000
H 4.59923900<br>0.19881400 -0.74771300	H -0.90804900<br>5.30929300 -0.04975400	H -5.44620000<br>0.50590000 -1.51700000	H 10.88290000<br>3.53630000 7.33810000
C -1.14351200<br>0.41722600 2.31808800	H -0.76103100<br>4.00818800 1.15042100	C 0.16830000<br>0.48520000 1.80710000	C 14.61590000<br>4.21250000 2.06230000
C -0.83968100 -<br>0.63846000 3.16028500	C 5.10705800<br>1.84658000 0.57761100	C -0.17700000<br>1.57780000 2.58310000	C 13.65510000<br>3.64980000 1.26480000
H -1.31089900 -<br>0.85770500 4.10798000	C 5.31910800<br>1.89576600 1.94071600	H 0.26020000<br>1.85330000 3.53240000	H 13.78270000<br>3.26730000 0.42620000
C 0.20030100 -<br>1.35760800 2.53228600	H 6.25987900<br>2.08829400 2.43778500	C -1.20530000<br>2.24760000 1.88490000	C 12.44670000<br>3.75920000 1.95330000
C -2.16614800<br>1.50011400 2.46240700	C 4.06407300<br>1.63389300 2.54097800	C 1.20200000 -<br>0.57190000 2.03780000	C 16.07070000<br>4.40050000 1.84290000
H -2.88258700<br>1.49491600 1.63310100	C 6.07462000<br>2.01040600 -0.55267800	H 1.95630000 -<br>0.58130000 1.24270000	H 16.26070000<br>5.33560000 1.74380000
H -2.72336300<br>1.34963100 3.39036200	H 6.13323000<br>1.10793300 -1.17341600	H 1.71180000 -<br>0.37950000 2.98530000	H 16.33700000<br>3.93340000 1.04810000
H -1.70852600<br>2.49640800 2.50910000	H 7.07255200<br>2.19990700 -0.14841600	H 0.76110000 -<br>1.57550000 2.09820000	H 16.55450000<br>4.05310000 2.59500000
C 0.91183700 -<br>2.59043000 3.00031700	H 5.81982400<br>2.85541500 -1.20524600	C -1.94870000<br>3.49240000 2.26330000	C 11.09040000<br>3.29250000 1.53300000

H 1.38523600 - 2.42746100 3.97545100	C 3.73726200 1.57786400 4.00243700	H -2.44350000 3.37790000 3.23490000	H 10.92570000 2.42020000 1.89820000
H 0.21501400 - 3.42950800 3.11120000	H 3.77583400 2.57605300 4.45685500	H -1.26960000 4.34970000 2.34060000	H 11.04910000 3.25180000 0.57470000
H 1.68697800 - 2.88133200 2.28608500	H 4.45511100 0.94750300 4.54027700	H -2.71100000 3.72650000 1.51530000	H 10.42580000 3.90500000 1.85550000
C -0.23490600 1.13150300 0.04631700	H 2.73462000 1.17534800 4.15617900	C -0.65620000 - 0.35960000 -0.45150000	C 14.51680000 5.33140000 4.33400000
H -0.93495100 1.95038800 0.19985000	C 3.05389800 1.36033100 -0.82709800	H 0.03940000 - 1.16660000 -0.22970000	H 15.48900000 5.22730000 4.26740000
C -0.65232900 0.34973400 -1.27013900	H 3.79309800 1.46220400 -1.62037900	C -0.19210000 0.36030000 -1.79070000	C 14.25290000 6.86160000 4.37210000
H -0.53729800 1.13829000 -2.04848200	C 2.42636200 - 0.07428800 -0.97444300	H -0.28810000 - 0.46150000 -2.53730000	H 14.81310000 7.22170000 5.09090000
C -2.15771800 0.04992300 -1.17189000	H 2.14628600 - 0.10728100 -2.04255500	C 1.31070000 0.65680000 -1.65320000	C 14.73110000 7.51180000 3.08920000
C -2.60540200 - 1.25383200 -0.93828200	C 3.54288700 - 1.10475300 -0.78042400	C 1.75440000 1.97310000 -1.50020000	C 13.95760000 7.50670000 1.93160000
H -1.86542500 - 2.04304800 -0.85630200	C 3.72948100 - 1.74041100 0.45292200	H 1.01280000 2.76460000 -1.51440000	H 13.14500000 7.05350000 1.92350000
C -3.96848100 - 1.53424400 -0.84454200	H 3.05360300 - 1.50169700 1.26711900	C 3.11380000 2.25520000 -1.36840000	C 14.38300000 8.16530000 0.79720000
H -4.29280700 - 2.55837500 -0.66850700	C 4.76288800 - 2.65962300 0.62945900	H 3.43710000 3.28880000 -1.25780000	H 13.85730000 8.13360000 0.02990000
C -4.93166200 - 0.52421500 -0.98359300	H 4.89399500 - 3.13639100 1.59926600	C 4.07550000 1.23500000 -1.38730000	C 15.57660000 8.87630000 0.77220000
C -4.48007400 0.77884500 -1.23238700	C 5.63973200 - 2.98186800 -0.41729500	C 3.62820000 - 0.08190000 -1.55770000	C 16.34310000 8.87360000 1.92490000
H -5.20465200 1.57934000 -1.36830100	C 5.44638600 - 2.34773100 -1.65020000	H 4.35370000 - 0.89190000 -1.60270000	H 17.14960000 9.33590000 1.93850000
C -3.11505600 1.05991700 -1.32738500	H 6.10392200 - 2.58602500 -2.48391600	C 2.26720000 - 0.36490000 -1.69140000	C 15.92980000 8.19560000 3.05680000
H -2.79641700 2.07845800 -1.54925400	C 4.41147000 - 1.42636500 -1.82952500	H 1.95280000 - 1.39630000 -1.85410000	H 16.47180000 8.20030000 3.81240000
C -6.40661300 - 0.83423500 -0.86445100	H 4.27081400 - 0.96537800 -2.80661600	C 5.54550000 1.54810000 -1.22380000	C 16.02980000 9.61640000 -0.45960000
H -7.02082600 - 0.01938200 -1.26179600	C 6.73652700 - 4.00429700 -0.22546100	H 6.17320000 0.71900000 -1.56760000	H 16.68810000 10.27000000 -0.21540000
H -6.66914300 - 1.74992400 -1.40686900	H 7.51810200 - 3.90177100 -0.98585600	H 5.83060000 2.44270000 -1.78940000	H 15.27830000 10.05510000 -0.86380000
H -6.70081600 - 0.98688200 0.18276400	H 6.34615100 - 5.02871000 -0.29497700	H 5.80140000 1.73820000 -0.17250000	H 16.41170000 8.99460000 -1.08380000

C 3.15830900 -	H 7.20853000 -	C -4.06400000	C 9.80740000
2.69395800 -0.48597400	3.90684700 0.75905400	3.36240000 -1.37610000	5.82550000 4.06350000
H 2.69874700 -	C -0.00001400	H -3.66670000	H 9.64910000
3.64413400 -0.17338700	1.20821200 3.20185200	4.33830000 -1.05560000	4.89110000 3.85510000
H 3.88246500 -	H 0.67688800	H -4.85590000	H 9.04190000
2.44951000 0.30727300	1.96844700 3.61784700	3.12160000 -0.65060000	6.16280000 4.55420000
C 3.91175000 -	H -1.00724400	C -4.69620000	C 9.99590000
2.91523900 -1.81018300	1.64544200 3.28946600	3.50490000 -2.77290000	6.62440000 2.77470000
H 4.42678000 -	C 0.06924000 -	H -5.14730000	H 10.11520000
2.00443600 -2.14575800	0.05925900 4.07338400	2.56330000 -3.11100000	7.55300000 2.98780000
H 4.67906900 -	H -0.62218200 -	H -5.48840000	H 9.22000000
3.70365200 -1.74209600	0.83754200 3.71886800	4.26960000 -2.80610000	6.52150000 2.21840000
H 3.23006600 -	H -0.17441700	H -3.95410000	H 10.76920000
3.20462000 -2.62066500	0.12104100 5.13414800	3.78740000 -3.53240000	6.30060000 2.30700000
	H 1.06936900 -		Zn 12.69560000
	0.51418400 4.05234600		8.46990000 6.35960000
	Zn -0.48577800 -		N 11.29230000
	0.96898100 -1.20074600		9.71770000 5.48050000
	N -1.04693800 -		N 10.00940000
	2.63060500 0.19433300		9.78960000 5.94410000
	N -2.07700500 -		N 11.51690000
	2.42576900 1.06160100		10.10850000 8.38640000
	N -3.13246800 -		N 10.18180000
	1.43178100 -1.60925800		9.84970000 8.33440000
	N -3.77380500 -		O 11.19460000
	1.57286800 -0.41754100		7.24130000 6.85080000
	O -1.32057700		C 9.22710000
	0.29779300 0.18091500		10.51400000 5.09320000
	C -2.08733100 -		C 10.04210000
	3.37048800 2.04986700		10.90320000 4.06460000
	C -1.01449800 -		H 9.79510000
	4.20689200 1.79810500		11.40860000 3.32410000
	H -0.71694900 -		C 11.30880000
	5.06457300 2.38465100		10.39970000 4.33800000
	C -0.39532800 -		C 7.78950000
	3.71045500 0.63105100		10.81010000 5.34610000
	C -3.09525600 -		H 7.68830000
	3.41248400 3.15612700		11.17510000 6.22780000
	H -4.11700000 -		H 7.47490000
	3.56223300 2.78551700		11.44510000 4.69820000
	H -2.86224900 -		H 7.27880000
	4.24577100 3.82389600		10.00060000 5.27410000

	H -3.08630600 -		C 12.59480000
	2.49479900 3.75649700		10.55970000 3.59590000
	C 0.81774100 -		H 12.89480000
	4.22609400 -0.08086800		9.70170000 3.28720000
	H 1.73343600 -		H 12.46080000
	3.76067700 0.30090800		11.14120000 2.84380000
	H 0.90783900 -		H 13.25690000
	5.30949600 0.04803500		10.93910000 4.17990000
	H 0.76115600 -		C 9.52390000
	4.00774600 -1.15148600		10.26290000 9.45570000
	C -5.10724900 -		C 10.48470000
	1.84587100 -0.57834900		10.82560000 10.25320000
	C -5.31929600 -		H 10.35720000
	1.89432900 -1.94148200		11.20810000 11.09180000
	H -6.26010800 -		C 11.69310000
	2.08638900 -2.43865500		10.71620000 9.56460000
	C -4.06418800 -		C 8.06910000
	1.63246300 -2.54159700		10.07490000 9.67510000
	C -6.07486200 -		H 7.87910000
	2.00998800 0.55185500		9.13980000 9.77420000
	H -6.13325500 -		H 7.80280000
	1.10778500 1.17300800		10.54200000 10.46980000
	H -7.07283600 -		H 7.58540000
	2.19906200 0.14749800		10.42230000 8.92300000
	H -5.82027300 -		C 13.04940000
	2.85535600 1.20403800		11.18280000 9.98490000
	C -3.73732100 -		H 13.21420000
	1.57577500 -4.00301900		12.05520000 9.61980000
	H -3.77575600 -		H 13.09070000
	2.57376900 -4.45787800		11.22360000 10.94320000
	H -4.45521500 -		H 13.71400000
	0.94525800 -4.54061600		10.57040000 9.66240000
	H -2.73471100 -		C 9.62310000
	1.17308800 -4.15654000		9.14400000 7.18400000
	C -3.05401000 -		H 8.65090000
	1.36067000 0.82661100		9.24810000 7.25060000
	H -3.79323800 -		C 9.88690000
	1.46275500 1.61984100		7.61370000 7.14590000
	C -2.42625900		H 9.32680000
	0.07378800 0.97460500		7.25370000 6.42700000
	H -2.14587900		C 9.40880000
	0.10609000 2.04265700		6.96360000 8.42870000

	C -3.54275500<br>1.10445700 0.78151400<br>C -3.72990400<br>1.74052400 -0.45154200<br>H -3.05446700<br>1.50197800 -1.26615500<br>C -4.76329400<br>2.65990200 -0.62727200<br>H -4.89484600<br>3.13698300 -1.59686600<br>C -5.63956900<br>2.98192800 0.42003000<br>C -5.44567900<br>2.34737700 1.65263500<br>H -6.10276800<br>2.58548100 2.48675800<br>C -4.41078400<br>1.42582500 1.83114700<br>H -4.26970500<br>0.96449200 2.80801300<br>C -6.73633400<br>4.00454900 0.22905900<br>H -7.51747500<br>3.90197900 0.98989500<br>H -6.34577100<br>5.02889100 0.29855200<br>H -7.20893500<br>3.90736700 -0.75519600<br>C 0.00011000 -<br>1.20894200 -3.20191300<br>H -0.67762400 -<br>1.96858800 -3.61762800<br>H 1.00685500 -<br>1.64737200 -3.28919700<br>C -0.06758600<br>0.05815600 -4.07410500<br>H 0.62460400<br>0.83587600 -3.71985800<br>H 0.17608800 -<br>0.12295700 -5.13472600<br>H -1.06722100<br>0.51418600 -4.05350200		C 10.18230000<br>6.96870000 9.58640000<br>H 10.99490000<br>7.42190000 9.59450000<br>C 9.75690000<br>6.31010000 10.72080000<br>H 10.28250000<br>6.34170000 11.48800000<br>C 8.56330000<br>5.59910000 10.74580000<br>C 7.79680000<br>5.60180000 9.59310000<br>H 6.99020000<br>5.13950000 9.57950000<br>C 8.21000000<br>6.27980000 8.46120000<br>H 7.66800000<br>6.27510000 7.70550000<br>C 8.11000000<br>4.85900000 11.97750000<br>H 7.45170000<br>4.20540000 11.73340000<br>H 8.86160000<br>4.42020000 12.38180000<br>H 7.72810000<br>5.48080000 12.60180000<br>C 14.33240000<br>8.64990000 7.45440000<br>H 14.49070000<br>9.58420000 7.66290000<br>H 15.09790000<br>8.31260000 6.96380000<br>C 14.14400000<br>7.85100000 8.74330000<br>H 14.02470000<br>6.92240000 8.53020000<br>H 14.91980000<br>7.95390000 9.29960000<br>H 13.37060000<br>8.17480000 9.21090000

2. Polymerization mechanism

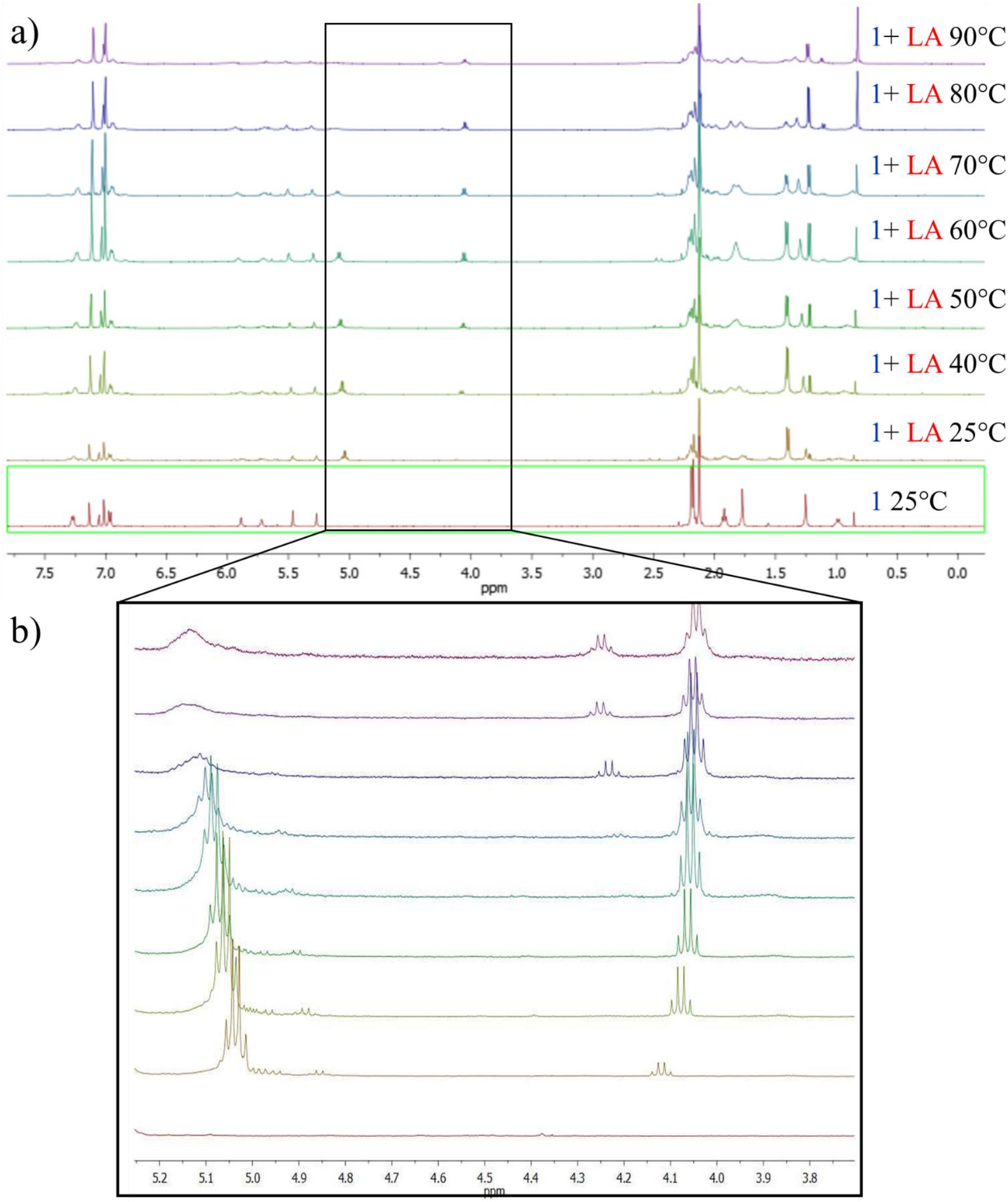

Figure S4: VT- ^{1}H NMR spectrum (500 MHz, 298 K, $CDCl_3$) of [LA]/[cat]=1. a) Full spectrum; b) CH region.

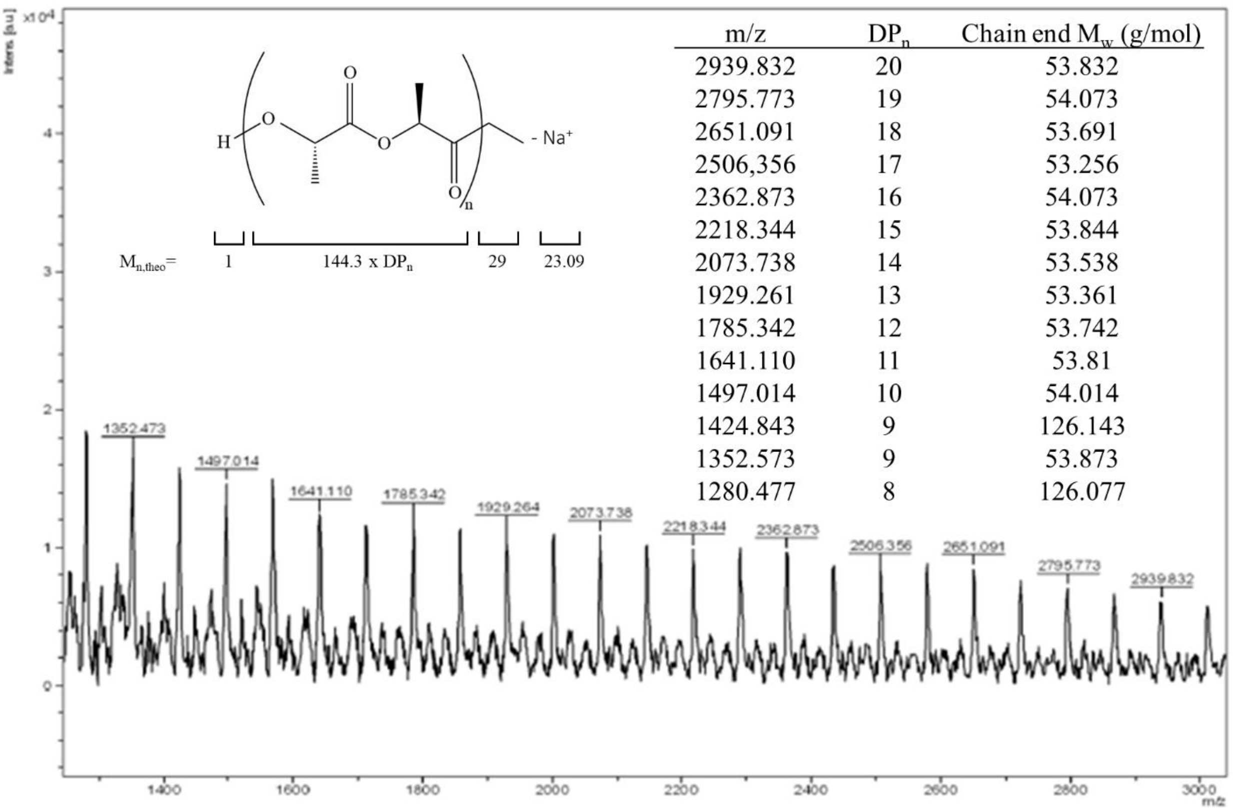

Figure S5. Selected area of MALDI-TOF mass spectrum of PLLA sample obtained with [LA]/[cat]=20, 79% conversion.

2.1 Calculation of P_m values of Racemic and stereo-diblock copolymers of PLA

Homodecoupled ^{1}H NMR spectra were obtained for racemic and stereo-diblock copolymers of PLA.

The methane region was deconvoluted and the curves were integrated using Origin software. The five tetrad mesodyads of the racemic spectrum were assigned according to literature (ovitt2002) and probability of each mesodyad was obtained after normalizing by the total area. Both, Bernouillan and non-Bernouillan statistics based on chain-end control mechanism (CEC) and enantiomorphic site control mechanism (ESC), respectively, were applied. For CEC mechanism, P_r was firstly obtained from

$$[rmr] = 0.5\ P_r^2 \qquad \text{Eq. S1}$$

to finally calculate P_m from

$$[mmm] = P_m^2 + 0.5\ P_m\, P_r \qquad \text{Eq. S2}$$

For ESC mechanism, P_m was directly obtained from

$$[mmm] = [P_m^2 + (1\text{-}P_m)^2 + P_m^3 + (1 - P_m)^3]/2 \qquad \text{Eq. S3}$$

Since the probabilities calculated using non-Bernouillan statistics remained unresolved, Bernouillan statistic was applied to the afforded stereo-diblock copolymers for calculation of P_m.

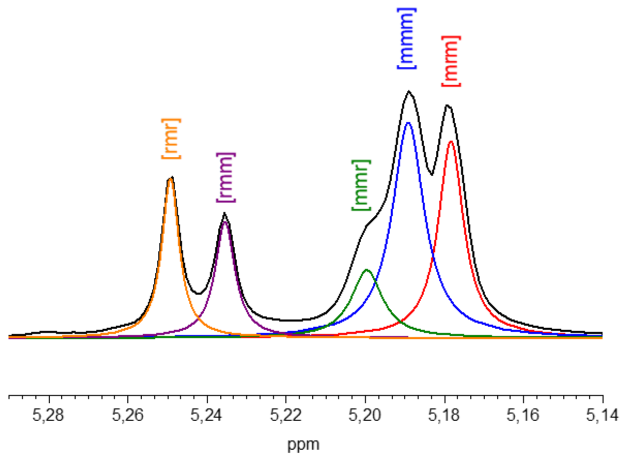

Figure S6. ^{1}H NMR spectra (500 MHz, 298 K, CDCl3,) of the homodecoupled CH resonance of poly(rac-lactide).

tetrad	Probability of ESC (non-Bernouillan)	Probability of CEC (Bernouillan)
mmm	$[P_m^2+(1-P_m)^2+P_m^3+(1-P_m)^3]/2$	$P_m^2+0.5\,P_mP_r$
mmr	$[P_m^2(1-P_m)+P_m(1-P_m)^2]/2$	$0.5\,P_mP_r$
rmm	$[P_m^2(1-P_m)+P_m(1-P_m)^2]/2$	$0.5\,P_mP_r$
rmr	$[P_m^2(1-P_m)+P_m(1-P_m)^2]/2$	$0.5\,P_r^2$
mrm	$[P_m(1-P_m)]$	$0.5\,(P_m^2+P_mP_r)$

Table S3. Tetrad probabilities of ESC and CEC mechanisms based on non-Bernouillan and Bernouillan statistics.

3. Polymerization kinetics

Entry	Sample	T (°C)	k_{app} x 10^2 (s^{-1})
1		90	6.59 ± 0.002
2	[a]PLLA400	75	2.56 ± 0.002
3		60	1.05 ± 0.0002
4	[b]PLLA500	90	5.61 ± 0.003
5	[b]PDLA500		4.71 ± 0.001

Table S4. Rate constants. [a] $[LA]_0/[Zn]_0$=400; $[LA]_0$= 400 mM; $[Zn]_0$= 1mM. [b]$[LA]_0/[Zn]_0$=500; [b]$[LA]_0$= 625 mM;$[Zn]_0$=1.25mM

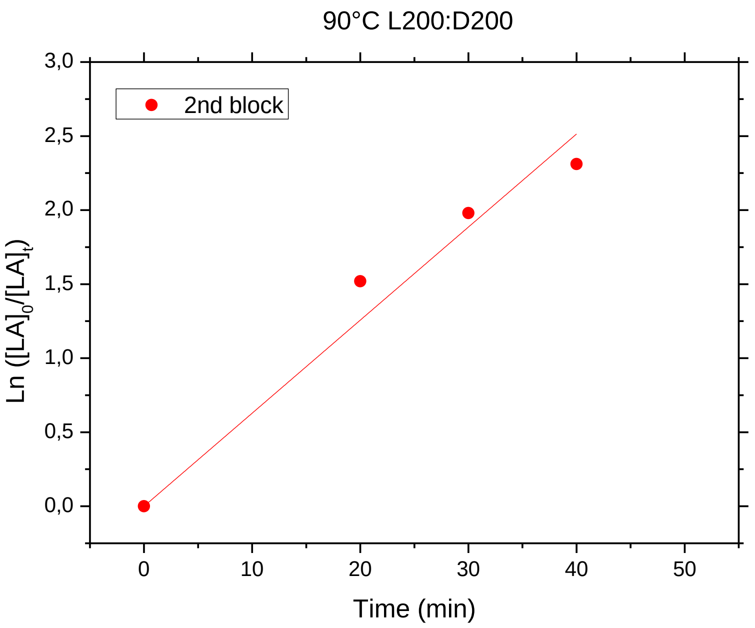

Figure S7. First-order kinetic plot of the second block polymerization reaction of L200:D200 at 90°C. $[D\text{-}LA]_0/[Zn]_0=200$; $[LA]_0= 200$ mM; $[Zn]_0= 1$mM.

4. Homochiral and racemic polymerization of lactide

4.1 Synthesis of L1: Homopolymer synthesis

Two schlenk tubes were charged in the glovebox with 10.4 mg and 360 mg of catalyst and L-LA respectively, and 2 and 3 mL of solvent respectively. Both, LA and catalyst schlenks flasks were attached to the vacuum line and temperature equilibrium was ensured by stirring the solutions for 15 min in an oil bath pre-heated at 70°C. Monomer and catalyst solutions were poured together by a glass bent adaptor and polymerization times were measured from that point. Methanol was used to terminate the reaction and precipitate the polymer synthesized after 60 min. The obtained polymer was collected by filtration and dried at room temperature exposed to vacuum over 24h, obtaining a white powder (97 mg, 27%). ^{1}H NMR (500 MHz, CDCl3, 297 K): δ 5.21-5.07 (q, 1H, $CH_{(PLA)}$), 4.99-4.94 (q, 1H, $CH_{(L\text{-}LA)}$), 1.62-1.60 (d, 3H, $CH_{3(L\text{-}LA)}$), 1.52-1.50 (d, 3H, $CH_{3(PLA)}$). GPC ($CHCl_3$): M_w=17440, M_n=12824, Đ=1.36.

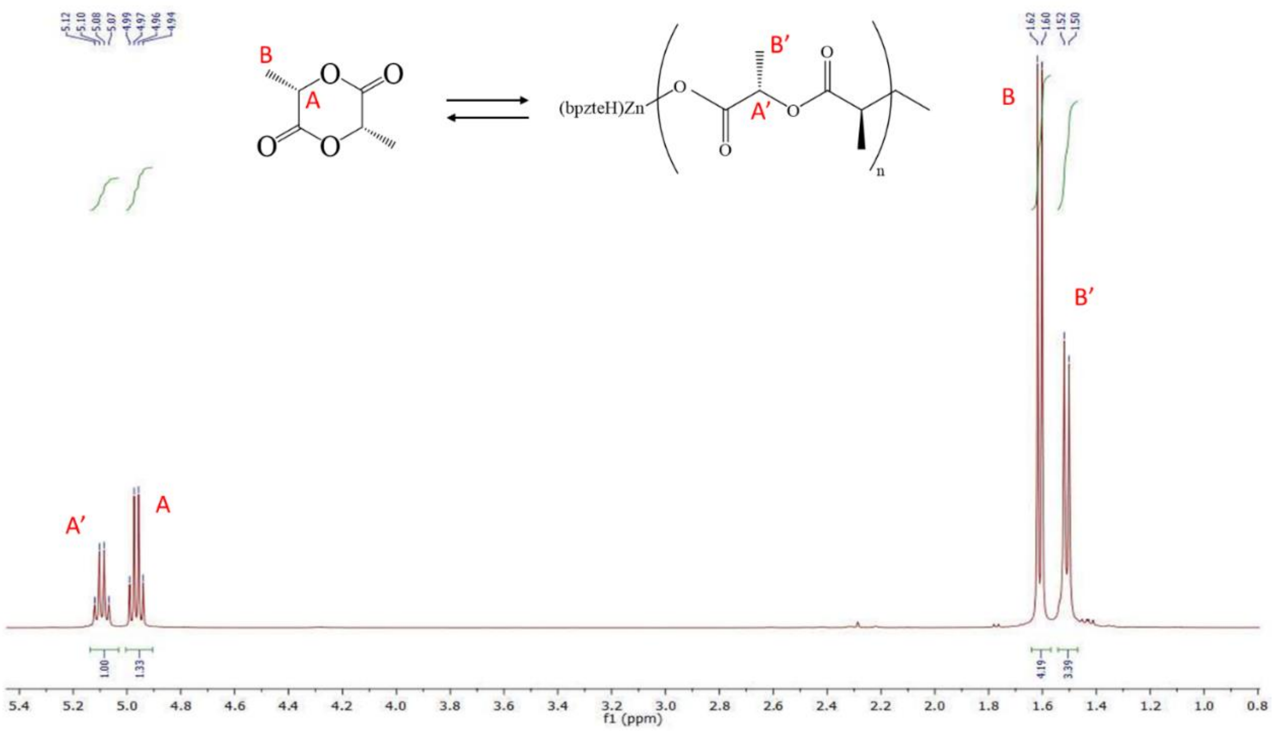

Figure S8: ^{1}H NMR spectrum (500 MHz, 298 K, $CDCl_3$) of L1.

4.2 Synthesis of L2

The polymer L2 was synthesized following the procedure described for the L1 polymer, namely the homopolymer synthesis. Two schlenk tubes were charged in the glovebox with 10.4 mg and 360 mg of catalyst and L-LA respectively, and 2 and 3 mL of solvent, respectively. The oil bath was pre-heated at 90°C rather than 70°C. A white powder was obtained after 60 min (270mg, 75%). ^{1}H NMR (500 MHz, CDCl3, 297 K): δ 5.21-5.16 (q, 1H, $CH_{(PLA)}$), 5.07-5.03 (q, 1H, $CH_{(L\text{-}LA)}$), 1.71-1.70 (d, 3H, $CH_{3(L\text{-}LA)}$), 1.61-1.59 (d, 3H, $CH_{3(PLA)}$). GPC ($CHCl_3$): M_w=48689, M_n=30055, Đ=1.62.

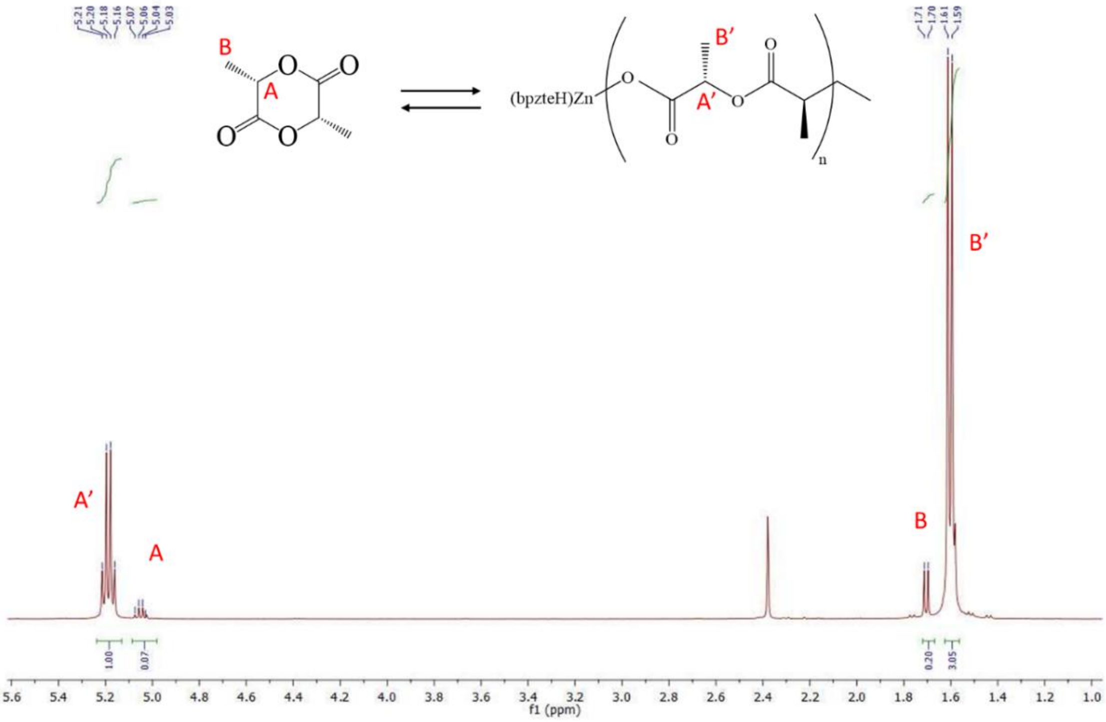

Figure S9: ^{1}H NMR spectrum (500 MHz, 298 K, $CDCl_3$) of L2.

4.3 Synthesis of L3

The polymer L3 was synthesized following the procedure described for the L1 polymer, namely the homopolymer synthesis. Two schlenk tubes were charged in the glovebox with 10.4 mg and 1.8 g of catalyst and L-LA respectively, and 5 and 15 mL of solvent respectively. The oil bath was pre-heated at 90°C. A white powder was obtained after 45 min (1.44g, 80%).^{1}H NMR (500 MHz, CDCl3, 297 K): δ 5.21-5.16 (q, 1H, $CH_{(PLA)}$), 5.07-5.02 (q, 1H, $CH_{(L\text{-}LA)}$), 1.71-1.70 (d, 3H, $CH_{3(L\text{-}LA)}$), 1.61-1.59 (d, 3H, $CH_{3(PLA)}$). GPC ($CHCl_3$): M_w=115118, M_n=62564, Đ=1.84.

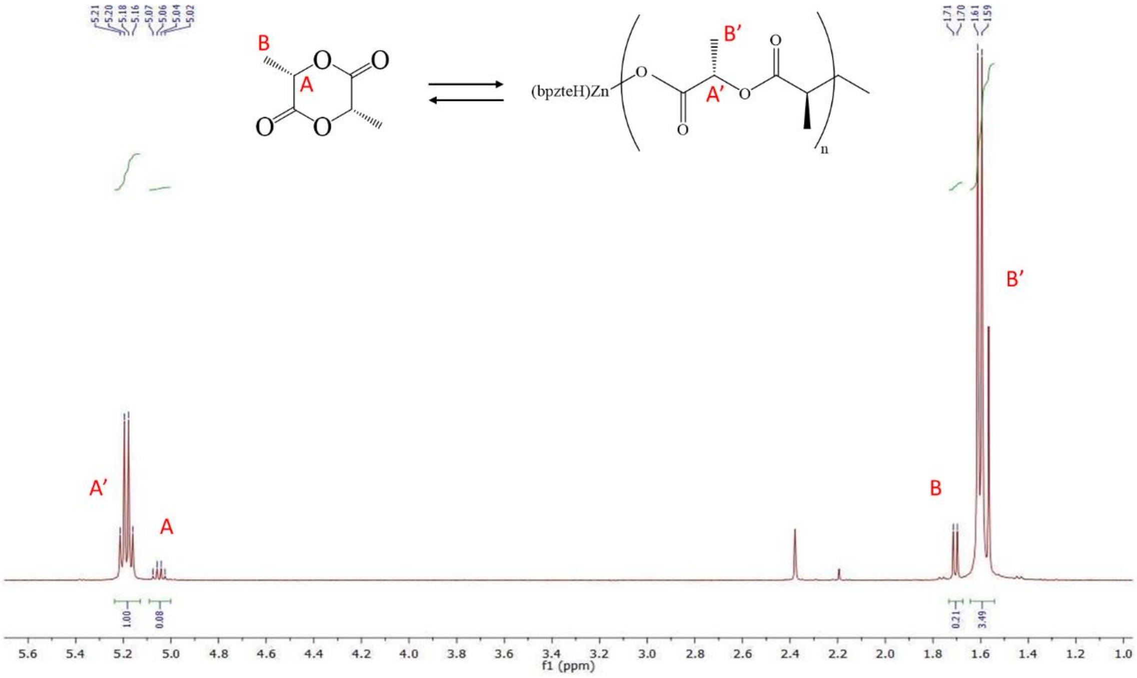

Figure S10: ^{1}H NMR spectrum (500 MHz, 298 K, $CDCl_3$) of L3**.**

4.4 Synthesis of L4

The polymer L4 was synthesized following the procedure described for the L1 polymer, namely the homopolymer synthesis. Two schlenk tubes were charged in the glovebox with 10.4 mg and 1.8 g of catalyst and L-LA and 5 and 15 mL of solvent, respectively. A white powder was obtained after 90 min (1.20g, 66 %).^{1}H NMR (500 MHz, CDCl3, 297 K): δ 5.21-5.16 (q, 1H, $CH_{(PLA)}$), 5.07-5.02 (q, 1H, $CH_{(L\text{-}LA)}$), 1.71-1.69 (d, 3H, $CH_{3(L\text{-}LA)}$), 1.61-1.59 (d, 3H, $CH_{3(PLA)}$). GPC ($CHCl_3$): M_w=75898, M_n=42880, Đ=1.77.

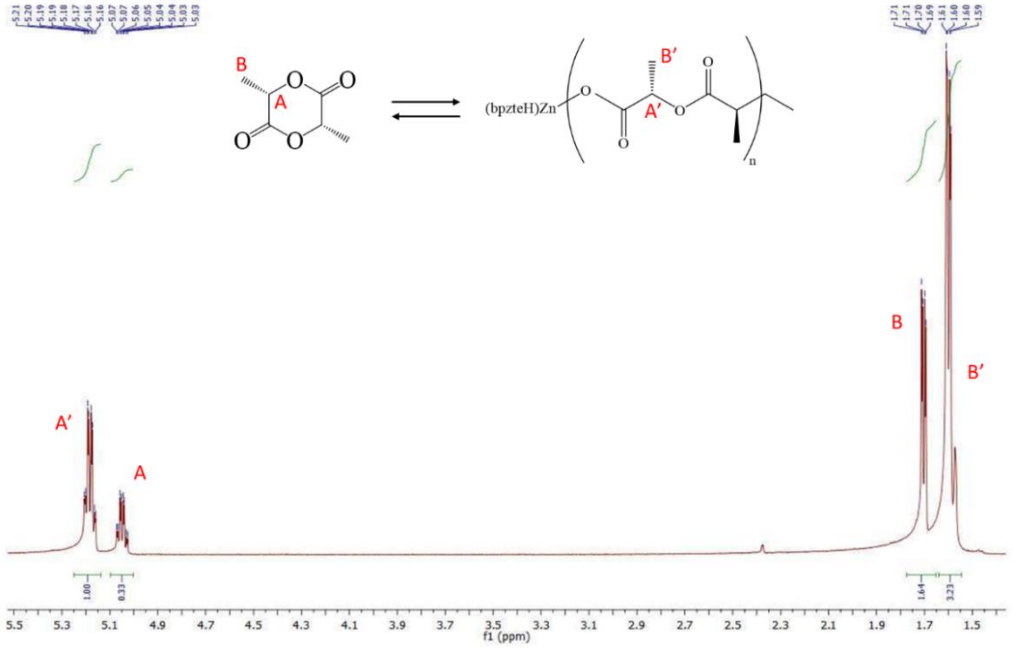

Figure S11: ^{1}H NMR spectrum (500 MHz, 298 K, $CDCl_3$) of L4.

4.5 Synthesis of L5

The polymer L5 was synthesized following the procedure described for the L1 polymer, namely the homopolymer synthesis. Two schlenk tubes were charged in the glovebox with 10.4 mg and 1.8 g of catalyst and L-LA and 5 and 15 mL of solvent, respectively. A white powder was obtained after 120 min (1.55g, 86 %). ^{1}H NMR (500 MHz, CDCl3, 297 K): δ 5.12-5.06 (q, 1H, $CH_{(PLA)}$), 4.98-4.93 (q, 1H, $CH_{(L\text{-}LA)}$), 1.62-1.60 (d, 3H, $CH_{3(L\text{-}LA)}$), 1.52-1.47 (d, 3H, $CH_{3(PLA)}$). GPC ($CHCl_3$): M_w=105673, M_n=48253, Đ=2.19.

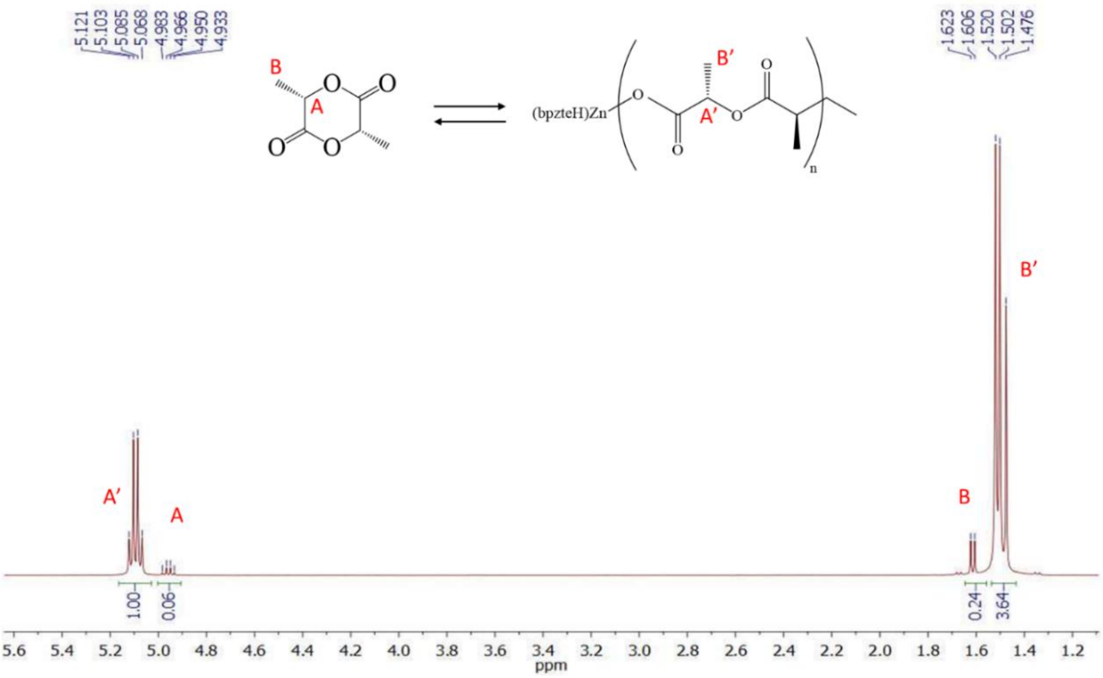

Figure S12: ^{1}H NMR spectrum (500 MHz, 298 K, $CDCl_3$) of L5.

4.6 Synthesis of D1

The polymer D1 was synthesized following the procedure described for the L1 polymer, namely the homopolymer synthesis. D-LA was used rather the L-LA. Two schlenk tubes were charged in the glovebox with 10.4 mg and 1.8 g of catalyst and D-LA and 5 and 15 mL of solvent, respectively. A white powder was obtained after 90 min (1.30g, 72 %). ^{1}H NMR (500 MHz, CDCl3, 297 K): δ 5.19-5.15 (q, 1H, $CH_{(PLA)}$), 5.06-5.02 (q, 1H, $CH_{(L\text{-}LA)}$), 1.70-1.69 (d, 3H, $CH_{3(L\text{-}LA)}$), 1.61-1.58 (d, 3H, $CH_{3(PLA)}$).GPC ($CHCl_3$): M_w=73500, M_n=41295, Đ=1.78.

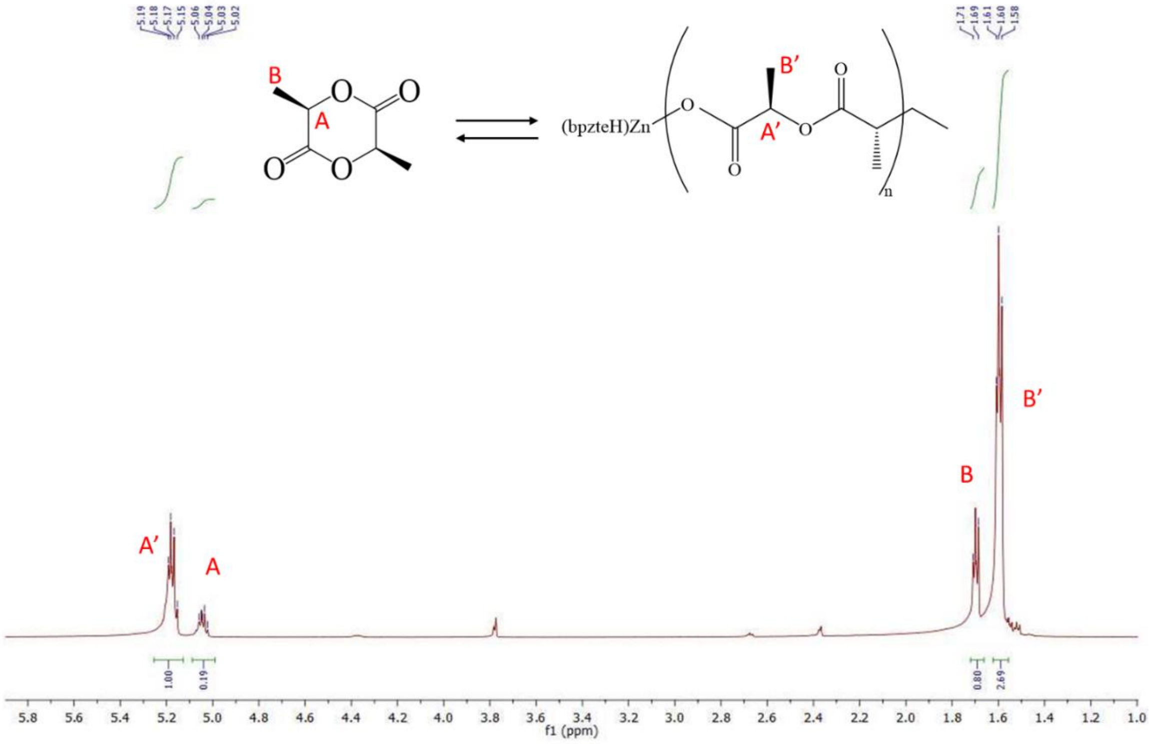

Figure S13: ^{1}H NMR spectrum (500 MHz, 298 K, $CDCl_3$) of D1.

4.7 Synthesis of Rac1

The polymer Rac1 was synthesized following the procedure described for the L1 polymer, namely the homopolymer synthesis. Racemic-LA (L-LA and D-LA) was used rather the L-LA. Two schlenk tubes were charged in the glovebox with 10.4 mg and 1.8 g of catalyst and Rac-LA and 5 and 15 mL of solvent, respectively. A transparent and rubbery product was obtained after 120 min (650 mg, 36 %). ^{1}H NMR (500 MHz, CDCl3, 297 K): δ 5.25-5.17 (m, 1H, $CH_{(PLA)}$), 5.16-5.05 (q, 1H, $CH_{(L-LA)}$), 1.70-1.68 (d, 3H, $CH_{3(L-LA)}$), 1.60-1.57 (m, 3H, $CH_{3(PLA)}$). GPC ($CHCl_3$): M_w=23592, M_n=14212, Đ=1.66.

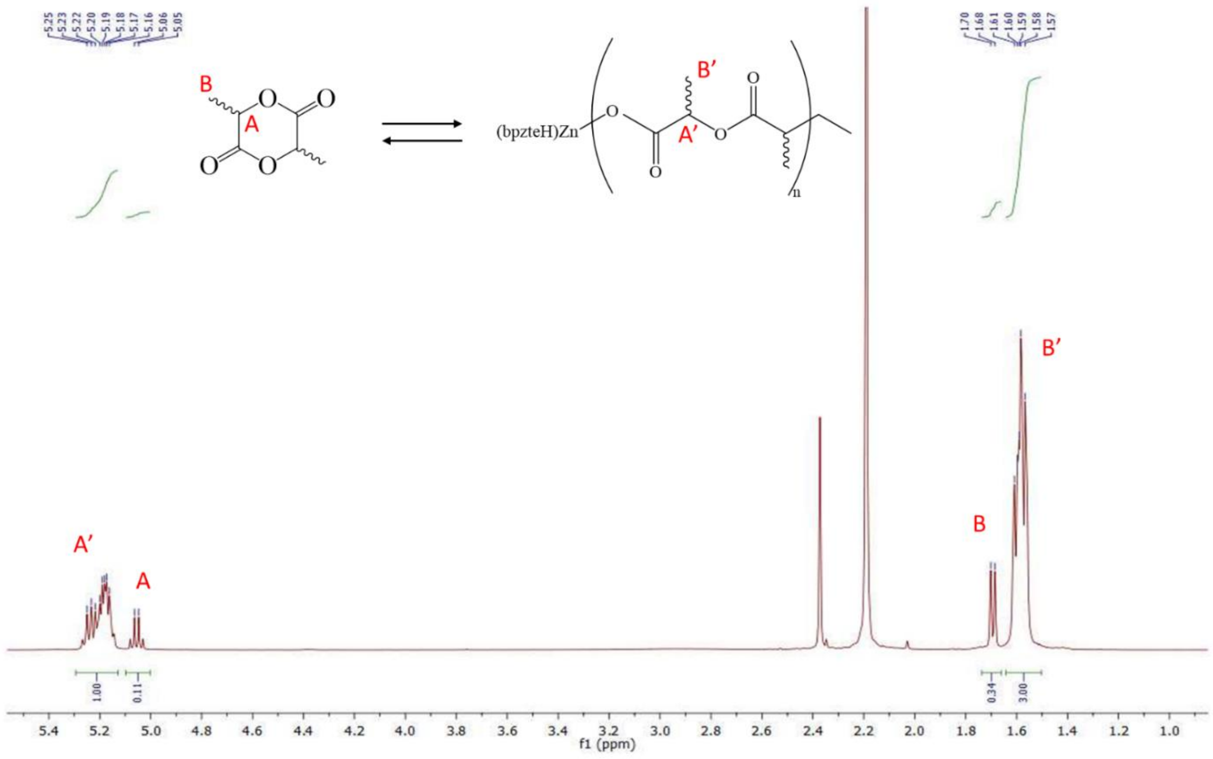

Figure S14. ^{1}H NMR spectrum (500 MHz, 298 K, $CDCl_3$) of Rac1.

5. Stereo-block copolymerization by sequential monomer addition

5.1 Synthesis of L50:D50: one pot sequential addition

The block-copolymer L50:D50 was synthesized following the one-pot sequential addition. Three schlenk tubes were charged in the glovebox with 10.4 mg, 180 mg and 180 mg of catalyst, L-LA and D-LA, respectively. 2 ml, 3ml and 3ml were added to the schlenk flasks respectively. Catalyst, L-LA and D-LA schlenks flasks were attached to the vacuum line and temperature equilibrium was ensured by stirring the solutions for 15 min in an oil bath pre-heated at 90°C. L-LA monomer and catalyst solutions were poured together by a glass bent adaptor and polymerization times were measured from that point. D-LA monomer solutions was added to the polymerization reaction after 25min of L-LA polymerization. Methanol was used to terminate the reaction and precipitate the polymer synthesized after 50 min. The obtained polymer was collected by filtration and dried at room temperature exposed to vacuum over 24h, obtaining a white powder (214 mg, 67%). ^{1}H NMR (500 MHz, CDCl3, 297 K): δ 5.21-5.16 (q, 1H, $CH_{(PLA)}$), 5.07-5.03 (q, 1H, $CH_{(L-LA)}$), 1.71-1.70 (d, 3H, $CH_{3(L-LA)}$), 1.61-1.59 (d, 3H, $CH_{3(PLA)}$). ^{13}C NMR (500 MHz, CDCl3, 297 K): δ 169.56 (CO), 68.95 (CH), 16.60 (CH_3). GPC ($CHCl_3$): M_w=32823, M_n=17007, Đ=1.93.

Figure S15: ^{1}H NMR spectrum (500 MHz, 298 K, $CDCl_3$) of L50:D50.

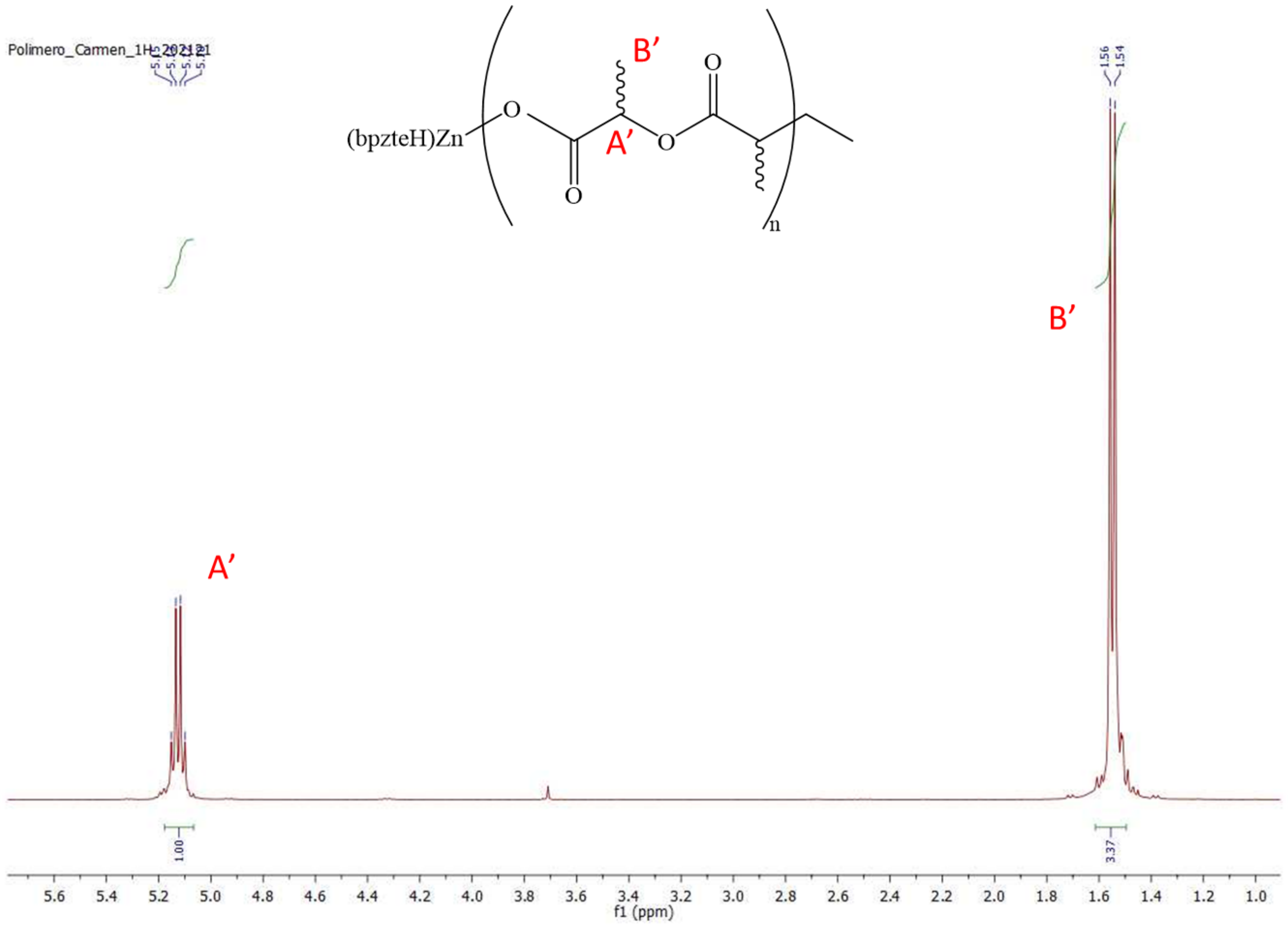

Figure S15: ^{1}H NMR spectrum (500 MHz, 298 K, $CDCl_3$) of L50:D50.

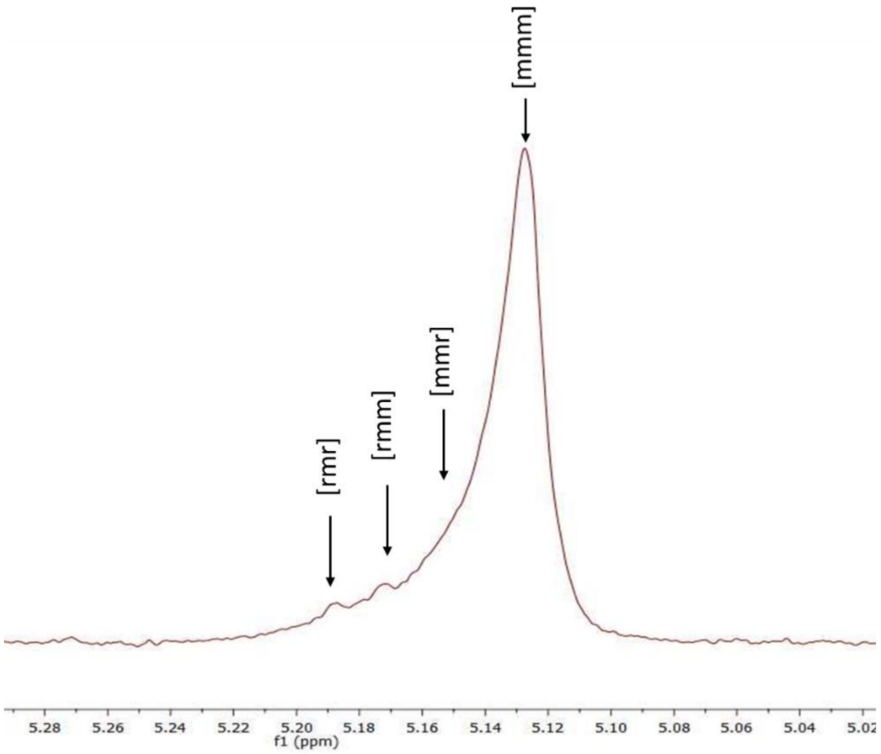

Figure S16: ^{1}H NMR spectrum (500 MHz, 298 K, $CDCl_3$) of the homodecoupled *CH* resonance of L50:D50.

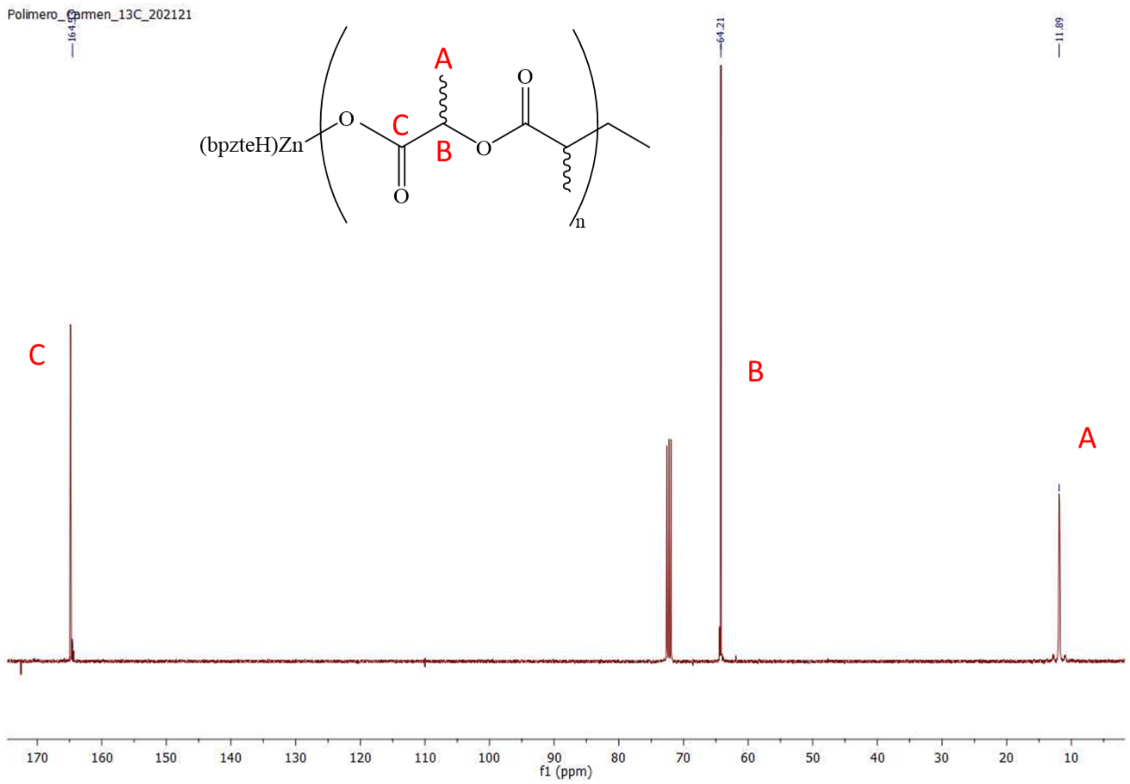

Figure S17: ^{13}C NMR spectrum (500 MHz, 298 K, $CDCl_3$) of L50:D50.

5.2 Synthesis of L100:D100

The block-copolymer L100:D100 was synthesized following the one-pot sequential addition. Three schlenk tubes were charged in the glovebox with 10.4 mg, 360 mg and 360 mg of catalyst, L-LA and D-LA, respectively. 5 ml, 7.5ml and 7.5ml were added to the schlenk flasks respectively. The second monomer solution (D-LA) was added after 30 min of L-LA polymerization. Methanol was used to terminate the reaction and precipitate the polymer synthesized after 60 min. The obtained polymer was collected by filtration and dried at room temperature exposed to vacuum over 24h, obtaining a white powder (587 mg, 83%). ^{1}H NMR (CDCl3, 297 K): δ 5.15-5.10 (q, 1H, $CH_{(PLA)}$), 5.02-4.98 (q, 1H, $CH_{(L-LA)}$), 1.66-1.64 (d, 3H, $CH_{3(L-LA)}$), 1.56-1.54 (d, 3H, $CH_{3(PLA)}$). ^{13}C NMR (CDCl3, 297 K): δ 169.56 (CO), 68.95 (CH), 16.60 (CH_3). GPC ($CHCl_3$): M_w=79320, M_n=35253, Đ=2.25.

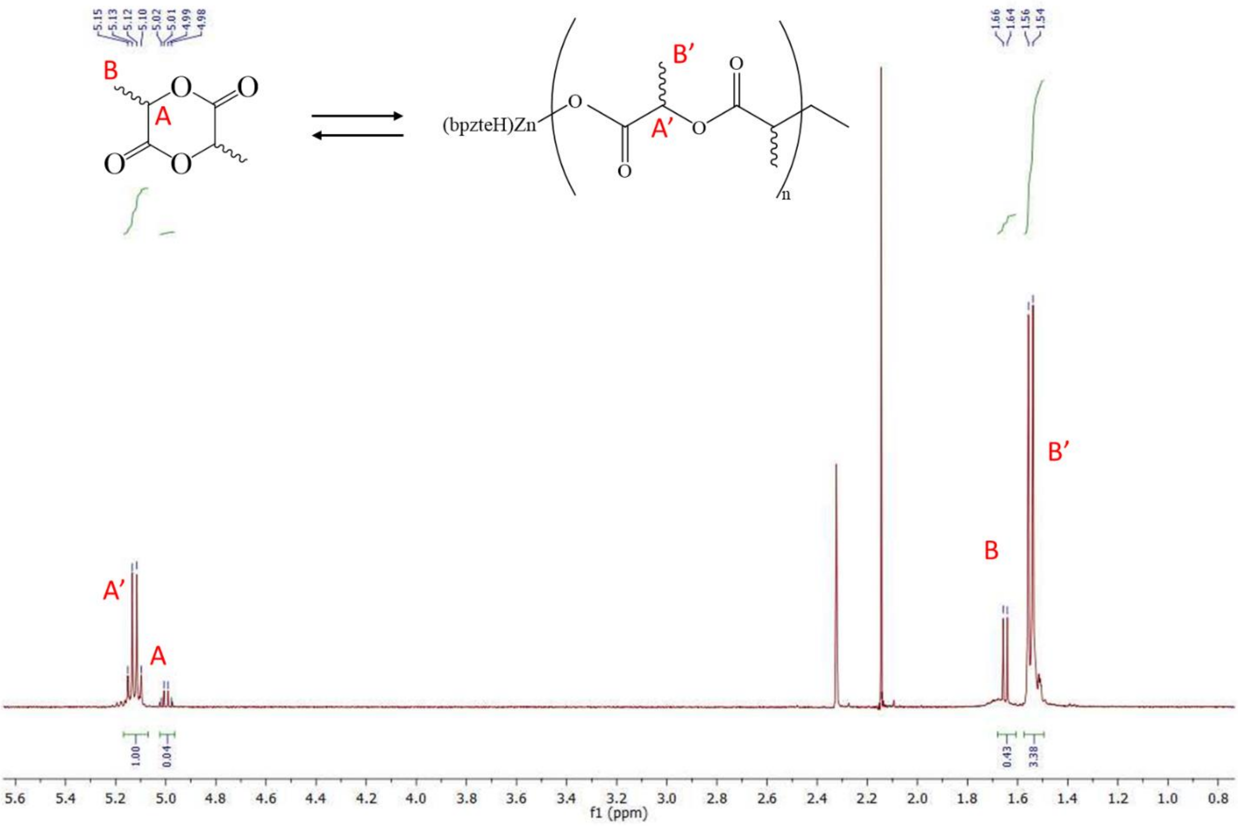

Figure S18: ^{1}H NMR spectrum (500 MHz, 298 K, CDCl3) of L100:D100.

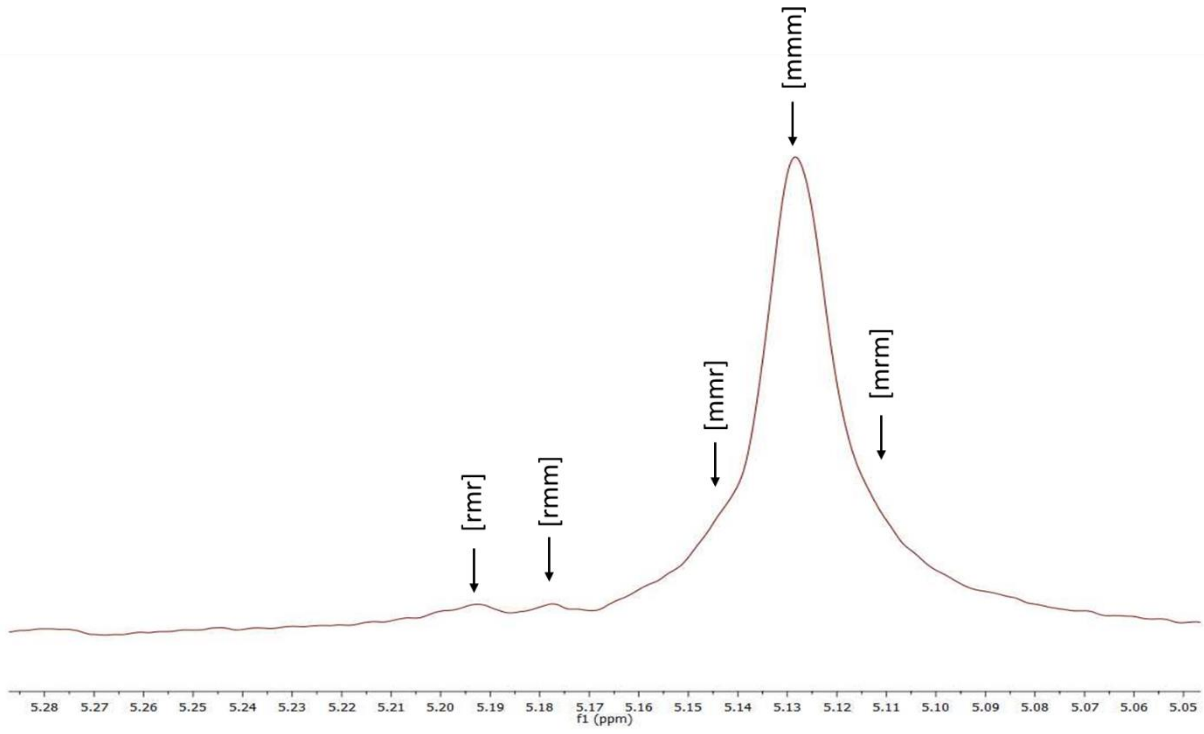

Figure S19: ^{1}H NMR spectrum (500 MHz, CDCl3, 297 K) of the homodecoupled *CH* resonance of L100:D100.

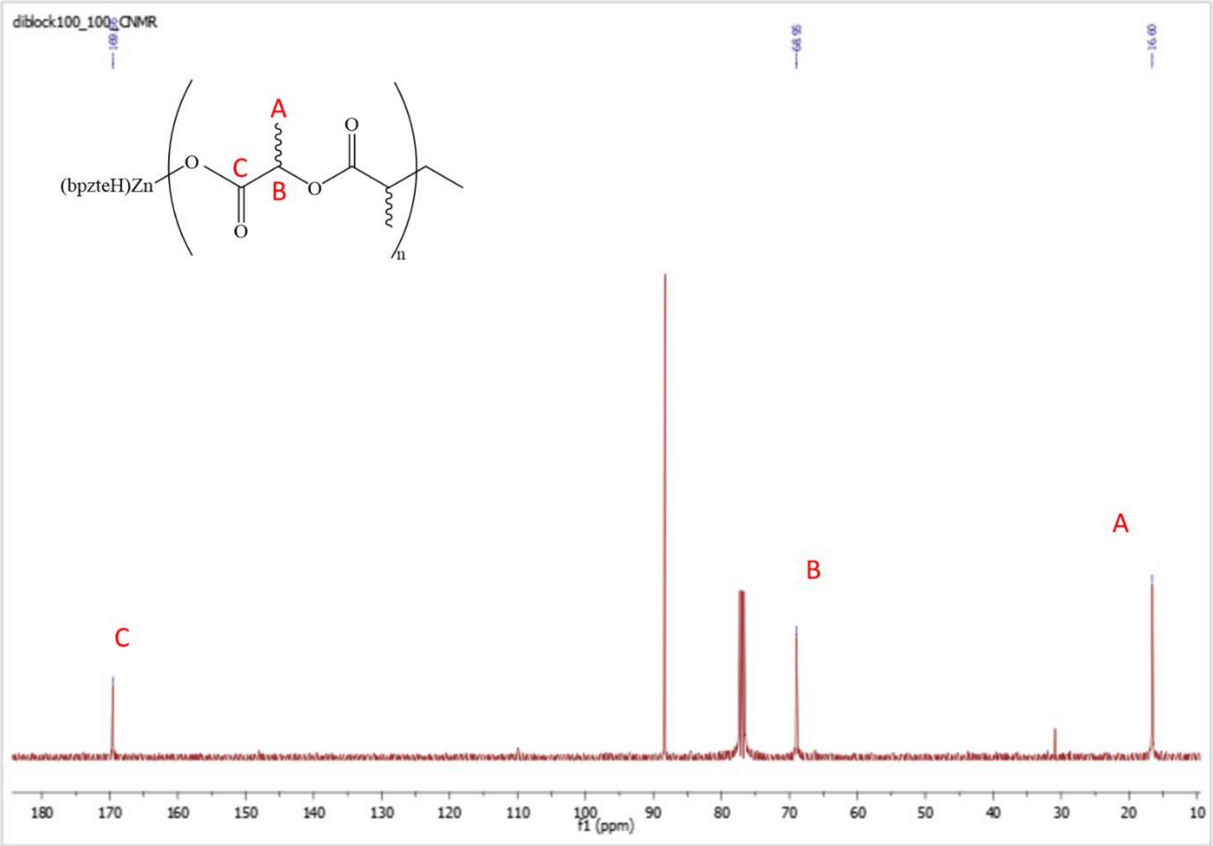

Figure S20: ^{13}C NMR spectrum (500 MHz, 298 K, $CDCl_3$) of L100:D100.

5.3 Synthesis of L300:D300

The block-copolymer L100:D100 was synthesized following the one-pot sequential addition. Three schlenk tubes were charged in the glovebox with 10.4 mg, 1,060 g and 1,060 mg of catalyst, L-LA and D-LA, respectively. 5 ml, 10ml and 10ml were added to the schlenk flasks respectively. The second monomer solution (D-LA) was added after 50 min of L-LA polymerization. Methanol was used to terminate the reaction and precipitate the polymer synthesized after 100 min. The obtained polymer was collected by filtration and dried at room temperature exposed to vacuum over 24h, obtaining a white powder (1,987 g, 92%). ^{1}H NMR (500 MHz, CDCl3, 297 K): δ 5.15-5.10 (q, 1H, $CH_{(PLA)}$), 5.02-4.98 (q, 1H, $CH_{(L-LA)}$), 1.66-1.64 (d, 3H, $CH_{3(L-LA)}$), 1.56-1.54 (d, 3H, $CH_{3(PLA)}$). ^{13}C NMR (500 MHz, CDCl3, 297 K): δ 169.56 (CO), 68.95 (CH), 16.60 (CH_3). GPC ($CHCl_3$): M_w=107912, M_n=63853, Đ=1.69.

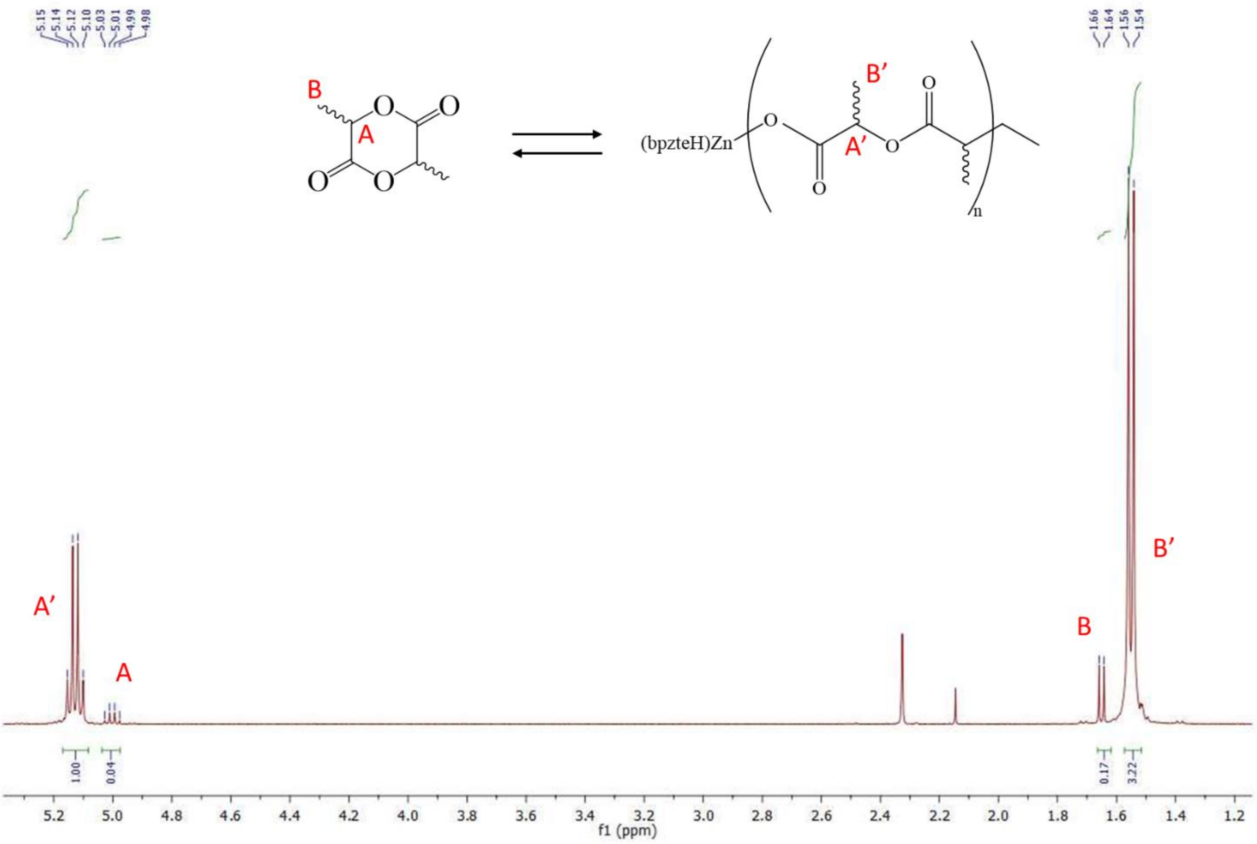

Figure S21: ^{1}H NMR spectrum (500 MHz, 298 K, $CDCl_3$) of L300:D300.

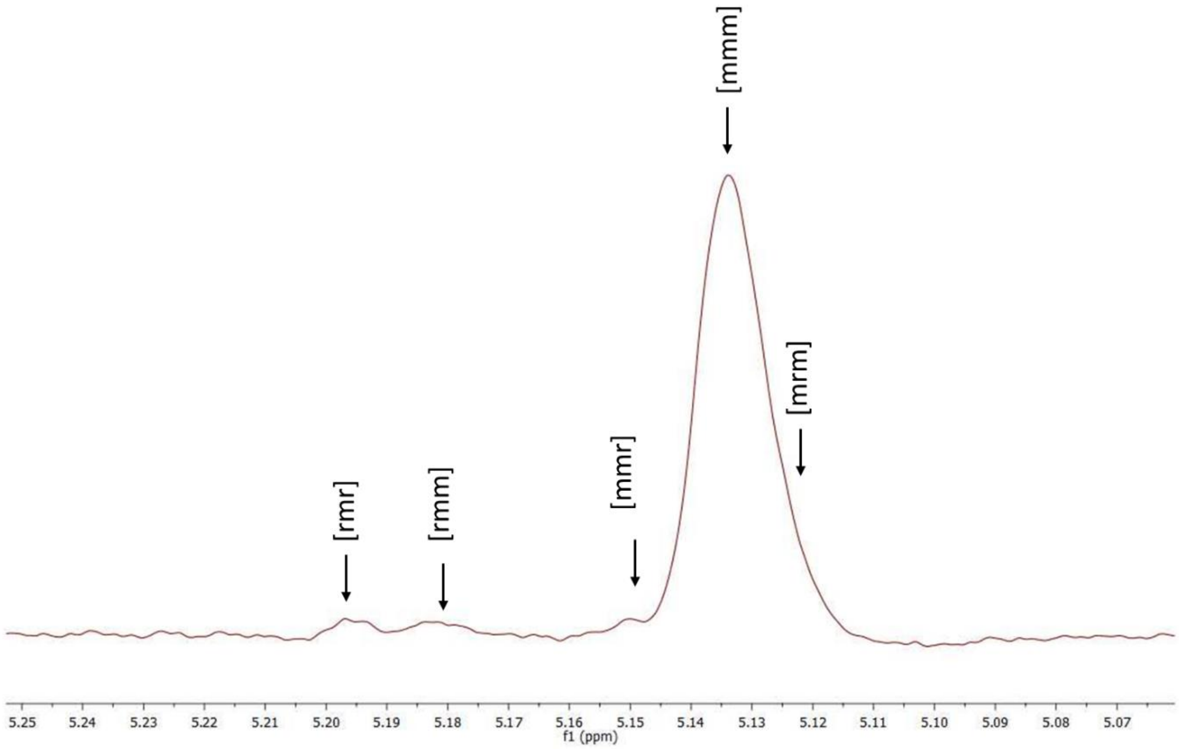

Figure S22: ^{1}H NMR spectrum (500 MHz, 298 K, $CDCl_3$) of the homodecoupled *CH* resonance of L300:D300.

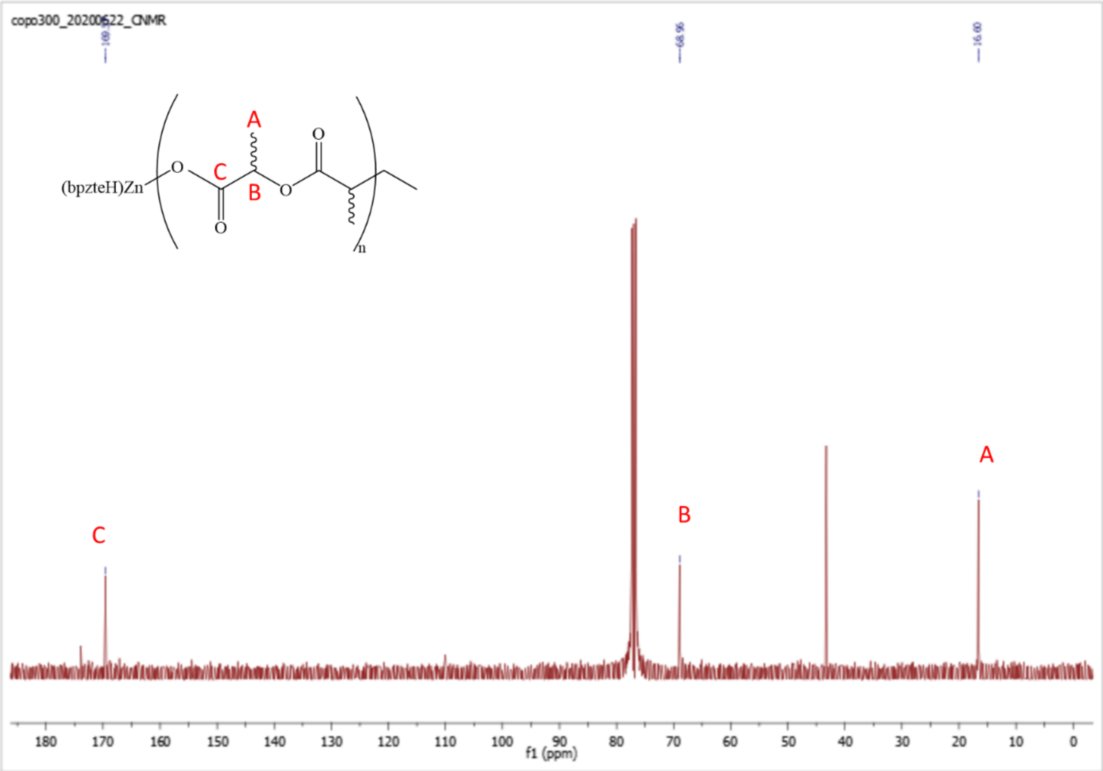

Figure S23: ^{13}C NMR spectrum (500 MHz, 298 K, $CDCl_3$) of L300:D300.

5.4 Synthesis of L500:D500

The block-copolymer L100:D100 was synthesized following the one-pot sequential addition. Three schlenk tubes were charged in the glovebox with 10.4 mg, 1,8 g and 1,8 mg of catalyst, L-LA and D-LA, respectively. 5 ml, 15ml and 15ml were added to the schlenk flasks respectively. The second monomer solution (D-LA) was added after 60 min of L-LA polymerization. Methanol was used to terminate the reaction and precipitate the polymer synthesized after 120 min. The obtained polymer was collected by filtration and dried at room temperature exposed to vacuum over 24h, obtaining a white powder (2,880 g, 80%). ^{1}H NMR (500 MHz, CDCl3, 297 K): δ 5.16-5.10 (q, 1H, $CH_{(PLA)}$), 5.02-4.99 (q, 1H, $CH_{(L-LA)}$), 1.66-1.64 (d, 3H, $CH_{3(L-LA)}$), 1.56-1.54 (d, 3H, $CH_{3(PLA)}$). ^{13}C NMR (500 MHz, CDCl3, 297 K): δ 169.56 (CO), 68.95 (CH), 16.60 (CH_3). GPC ($CHCl_3$): M_w=150094, M_n=64976, Đ=2.31. GPC ($CHCl_3$): M_w=150094, M_n=64976, Đ=2.31.

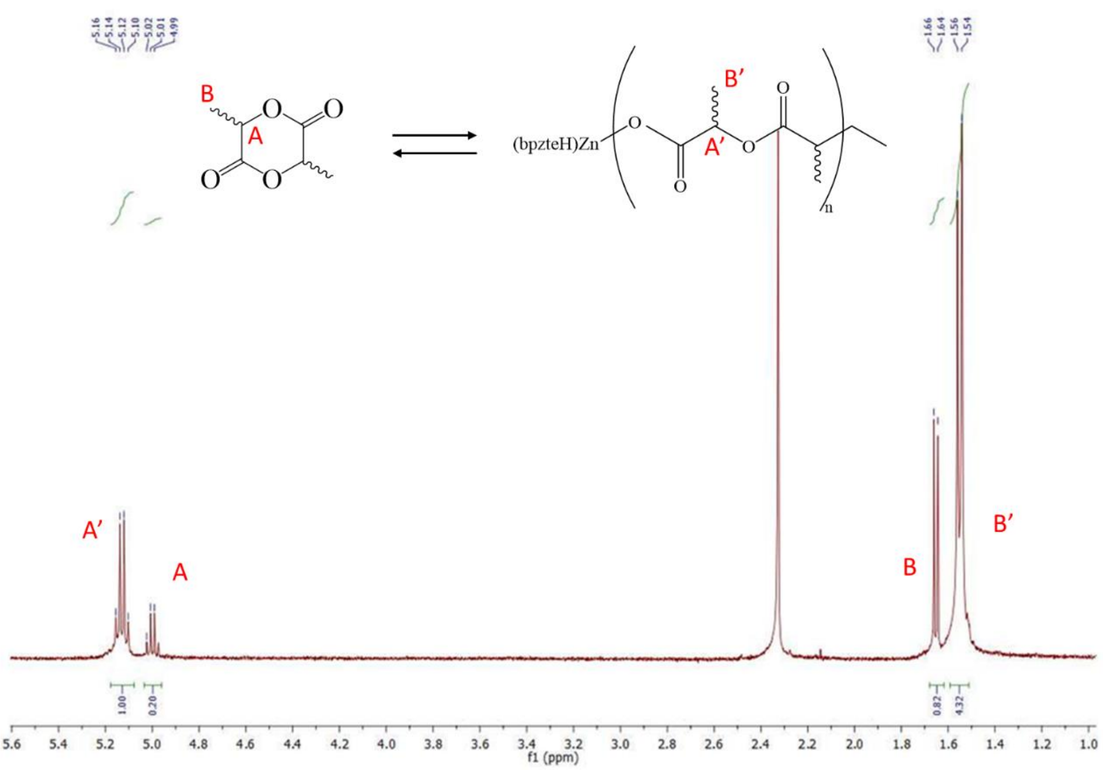

Figure S24: ^{1}H NMR spectrum (500 MHz, 298 K, $CDCl_3$) of L500:D500.

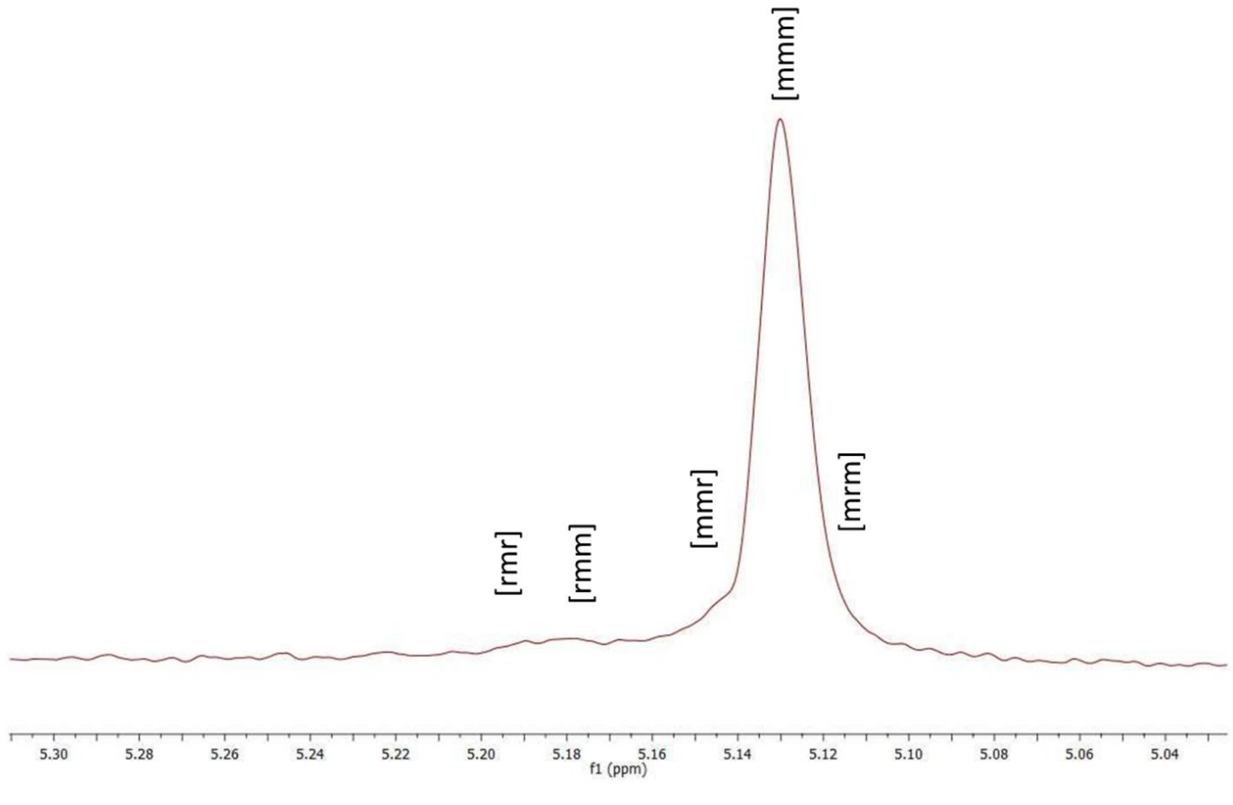

Figure S25: ^{1}H NMR spectrum (500 MHz, 298 K, $CDCl_3$) of the homodecoupled *CH* resonance of L500:D500.

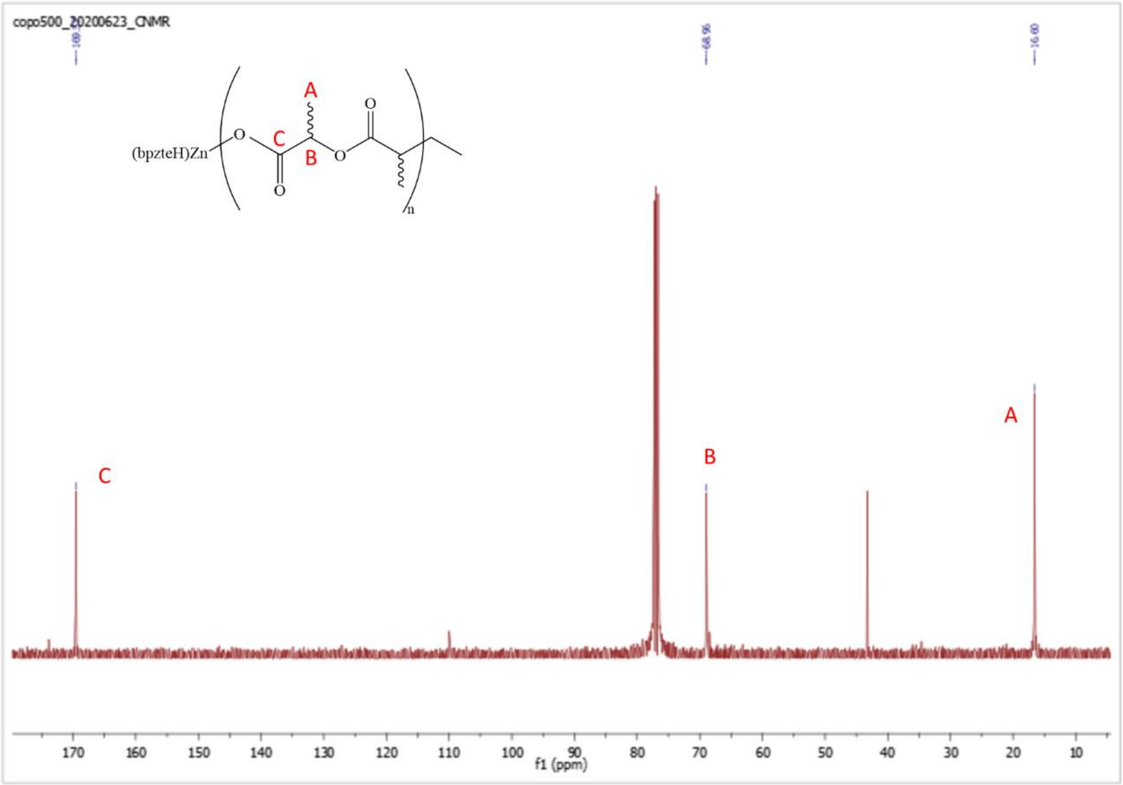

Figure S26: ^{13}C NMR spectrum (500 MHz, 298 K, $CDCl_3$) of L500:D500.

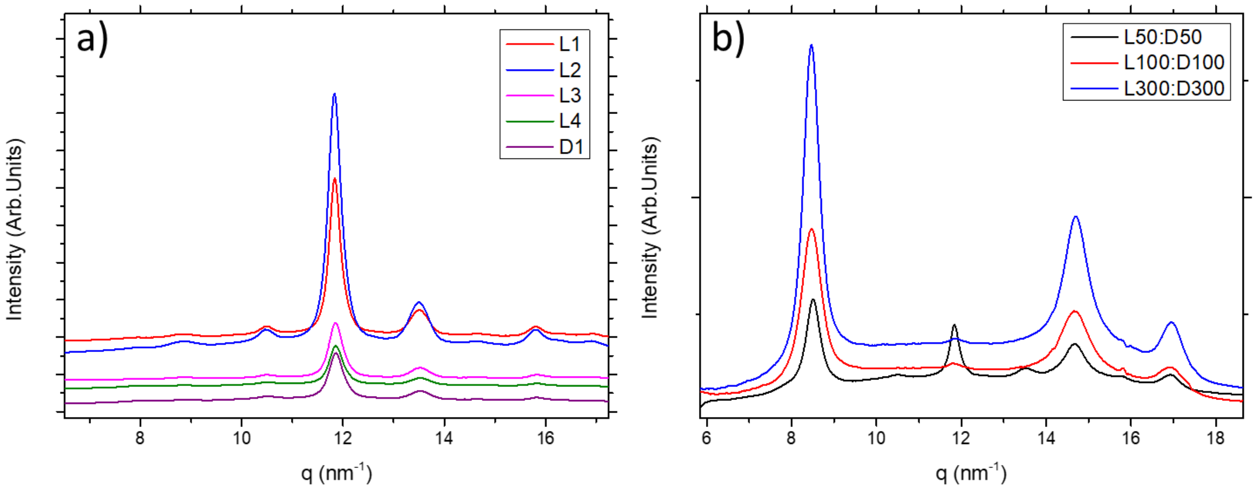

Figure S27: WAXS patterns acquired at room temperature. a) Homopolymers b) Stereo-diblock copolymers.
